# Using Instrumental Variables to Measure Causation over Time in Cross-Lagged Panel Models

**DOI:** 10.1080/00273171.2023.2283634

**Published:** 2024-02-15

**Authors:** Madhurbain Singh, Brad Verhulst, Philip Vinh, Yi (Daniel) Zhou, Luis F. S. Castro-de-Araujo, Jouke-Jan Hottenga, René Pool, Eco J. C. de Geus, Jacqueline M. Vink, Dorret I. Boomsma, Hermine H. M. Maes, Conor V. Dolan, Michael C. Neale

**Affiliations:** a Department of Human and Molecular Genetics, Virginia Commonwealth University; b Virginia Institute for Psychiatric and Behavioral Genetics, Virginia Commonwealth University; c Department of Biological Psychology, Vrije Universiteit Amsterdam; d Department of Psychiatry and Behavioral Sciences, Texas A&M University; e Department of Psychiatry, Virginia Commonwealth University; f Amsterdam Public Health Research Institute; g Behavioural Science Institute, Radboud University

**Keywords:** Causal inference, instrumental variables, CLPM, lagged effects

## Abstract

Cross-lagged panel models (CLPMs) are commonly used to estimate causal influences between two variables with repeated assessments. The lagged effects in a CLPM depend on the time interval between assessments, eventually becoming undetectable at longer intervals. To address this limitation, we incorporate instrumental variables (IVs) into the CLPM with two study waves and two variables. Doing so enables estimation of both the lagged (i.e., “distal”) effects and the bidirectional cross-sectional (i.e., “proximal”) effects at each wave. The distal effects reflect Granger-causal influences across time, which decay with increasing time intervals. The proximal effects capture causal influences that accrue over time and can help infer causality when the distal effects become undetectable at longer intervals. Significant proximal effects, with a negligible distal effect, would imply that the time interval is too long to estimate a lagged effect at that time interval using the standard CLPM. Through simulations and an empirical application, we demonstrate the impact of time intervals on causal inference in the CLPM and present modeling strategies to detect causal influences regardless of the time interval in a study. Furthermore, to motivate empirical applications of the proposed model, we highlight the utility and limitations of using genetic variables as IVs in large-scale panel studies.

## Introduction

Cross-lagged panel models (CLPM) are widely used to infer causal relationships between variables by analyzing observational data with repeated assessments. The magnitude and statistical significance of the lagged causal effects in the CLPM depend on the time interval between repeated assessments (Kuiper & Ryan, 2018). The lagged effect typically decays with an increasing time interval, which limits the reliable detection of causation in the CLPM. To address this limitation, we incorporate instrumental variables (IVs) into the CLPM (henceforth, IV-CLPM) to estimate both cross-sectional and lagged causal effects. In this paper, we focus on the impact of measurement intervals on the causal estimates in the traditional CLPM and the IV-CLPM, using both simulated and empirical data.

**Figure 1.**
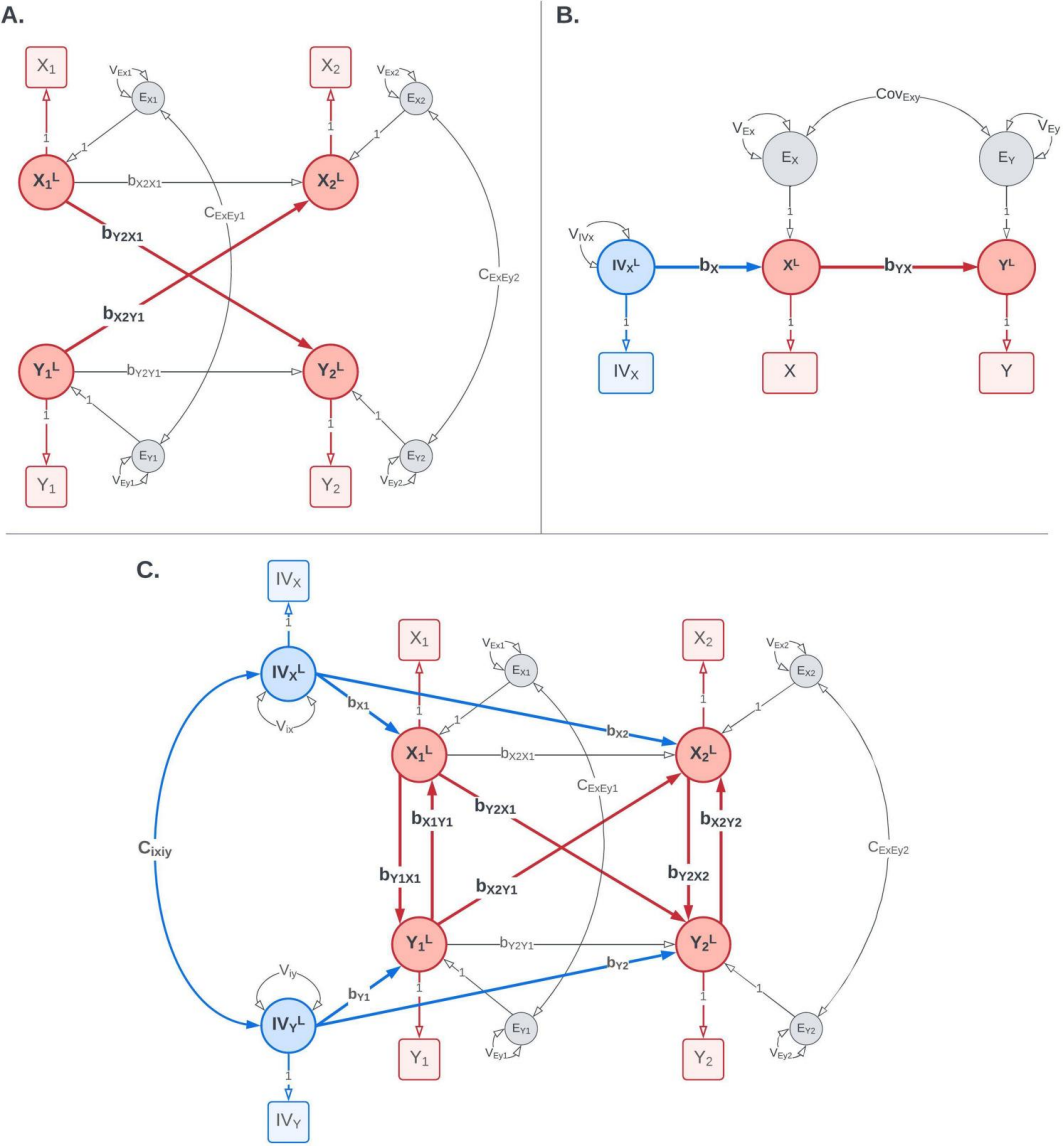
(A) CLPM: The cross-lagged panel model (CLPM) is used to estimate bidirectional lagged effects between *X* and *Y* ($b_{Y2X1}$ and $b_{X2Y1}$). This model was used as the reference model for integrating instrumental variables. (B) IV Regression: The instrumental variables regression (IVR) model fitted in a Structural Equation Modeling framework. The model uses the instrumental variable for *X*, *IVx*, to estimate the causal effect of *X* on *Y* ($b_{YX}$). (C) IV-CLPM: The proposed IV-CLPM model combines the CLPM with bidirectional IVR applied cross-sectionally at each wave. In addition to the lagged (i.e., “distal”) effects $b_{Y2X1}$ and $b_{X2Y1},$ the model utilizes IVR to estimate cross-sectional (i.e., “proximal”) effects at each wave: $b_{Y1X1}$ and $b_{X1Y1}$ at wave 1, and $b_{Y2X2}$ and $b_{X2Y2}$ at wave 2. In all three path diagrams, squares/rectangles represent the observed variables, and circles represent latent variables. To improve readability, the modeling of means is not shown in this figure. For complete path diagrams with means, please see Figure A1 in the Appendix A.

The CLPM is used to estimate causal influences over time based on Granger causality (Granger, 1969), wherein the cause temporally precedes the outcome and predicts the future values of the outcome. In the simplest CLPM (Figure 1(A)), two variables, say X and Y, are assessed on two occasions ($T_{1}$ and $T_{2}$). The model is considered “crossed” as it allows for the estimation of *bidirectional* causal influences between X and Y, and it is considered “lagged” as the effects are estimated across time (i.e., from $T_{1}$ to $T_{2}$). In addition, the CLPM controls for (i) the cross-sectional correlation between the residuals of X and Y on each occasion (subsuming the covariance due to omitted confounding variables), and (ii) autoregressive effects across time, indicating the degree of stability of each construct (X and Y) over time. The predictive causal inference in CLPM may help to identify potential targets for interventions when experimental designs are either infeasible or unethical. As a result, the CLPM has long been a particularly appealing approach in social and behavioral research (e.g., Becker et al., 2012; Hawkley et al., 2010; Lac & Donaldson, 2021; Patalay et al., 2015; van Ouytsel et al., 2019).

The lagged effects estimated in the CLPM depend on the time interval between repeated observations taken at times $T_{1}$ and $T_{2},$ i.e., $\Delta T=T_{2}-T_{1}$ (Kuiper & Ryan, 2018). In practice, the time scale of measurements depends on multiple factors, including the process being studied, the research design, and the feasibility of the time frame. It may range from milliseconds to months for neuroimaging, behavioral, and psychological traits and to years for maturational processes. Given an appropriate time scale, as the time interval between the predictor and the outcome increases, the lagged effect decays asymptotically (e.g., as seen with the influence of loneliness on blood pressure; Hawkley et al., 2010), ultimately becoming practically undetectable at longer time intervals. Consequently, the failure to reject the null hypothesis of no causation may be due to either the absence of a causal effect or an inappropriate time interval. For example, in a study of problem behaviors in early adolescence, Becker et al. (2012) found that delinquent behaviors predicted marijuana use, but not alcohol use, assessed nine months later. Here, the interpretation of the non-significant lagged effect of delinquent behaviors on alcohol use is ambiguous. One cannot distinguish whether delinquent behaviors do not have a causal effect on alcohol use, or whether the time interval is too long for this effect to be detected. Therefore, a lack of evidence for lagged effects at a given time interval cannot be generalized to the overall causal relationship between two traits.

Among alternative methods of causal inference with observational data, Instrumental Variables Regression (IVR) is increasingly popular in fields, such as economics (e.g., Cambini & Rondi, 2010; Hassan et al., 2020; Rakowski & Yamani, 2021) and epidemiology (e.g., George et al., 2022; Hamer et al., 2021; McDowell et al., 2015). This approach uses one or more exogenous predictors (i.e., the instrumental variables, IVs) of the hypothesized causal variable *X* to estimate its effect on the outcome *Y*. If the assumptions of IVR are met, the regression coefficient in the regression of *Y* on *X* represents the causal effect of *X* on *Y* (Bollen, 2012). Maydeu-Olivares et al. (2019) and Minică et al. (2018) have demonstrated that the IVR model can be implemented within the SEM framework using Maximum Likelihood estimation.

Although the IVR model allows for the estimation of causal effects in cross-sectional data, it does not necessarily imply that there is no temporal ordering of the cause and the outcome in the underlying causal process. This is because temporal precedence of the cause over the outcome has usually been accepted as a prerequisite for causality since David Hume’s seminal work in the philosophy of causation (Hume, [1739] 2009). For example, in a population-level study of the relationship between unemployment and mental health in Finland, Amin et al. (2023) demonstrated using IVR analyses that unemployment rates had a detrimental causal effect on self-reported mental health. Here, the IVR analyses allowed the authors to differentiate the causal effect of unemployment on mental health from other sources of covariance (e.g., omitted confounding variables or reverse causality), even though the two variables were assessed simultaneously. However, in the underlying causal process, unemployment (the cause) would still be expected to happen before a consequent decline in mental health (the outcome).

In this paper, we combine IVR with the CLPM by incorporating two IVs (one for each construct) in the traditional CLPM (henceforth, IV-CLPM). In addition to the lagged (i.e., “distal”) effects traditionally estimated in the CLPM, the IV-CLPM approach enables us to estimate cross-sectional (i.e., “proximal”) causal influences at each wave, without needing temporal ordering of the two variables. Using simulated data, we compared the proposed IV-CLPM and the traditional CLPM approaches and investigated how the time interval between repeated assessments impacts the causal inference in both models. We demonstrate that, while the distal effects become undetectable at longer time intervals, the causal relationship between *X* and *Y* remains detectable in the IV-CLPM in the two proximal effects (one at each wave). With the three causal effects estimated in the IV-CLPM, it is possible to test these parameter estimates separately or jointly using likelihood-ratio tests, which helps to evaluate different temporal aspects of causal influences. Furthermore, comparing these likelihood-ratio test statistics provides a novel way to examine whether the time interval in a study is appropriate for studying Granger-causal influences between variables.

To provide an empirical example, we examined the causal influences between cigarette smoking and alcohol consumption using genetic variants as IVs. Tobacco and alcohol are among the most commonly used legal substances of abuse (Hurley et al., 2012) and are leading contributors to the global disease burden (Gakidou et al., 2017). Epidemiological studies demonstrate a high degree of comorbidity between tobacco smoking and alcohol use (Mckee & Weinberger, 2013). However, simple regression models in observational data cannot differentiate the extent to which this comorbidity can be attributed to (i) the causal effects of smoking on alcohol use, (ii) the causal effects of alcohol use on smoking, and (iii) the effects of omitted variables influencing both smoking and alcohol use. The third category could include biological (e.g., genetic factors, brain reward pathways), psychological (e.g., externalizing and internalizing behaviors), and social (e.g., contextual cues) factors (Hurley et al., 2012). Moreover, evidence from experimental psychopharmacological studies suggests that the potential causal effects between smoking and alcohol use could plausibly be acute (i.e., proximal), chronic (i.e., distal), or both (Hurley et al., 2012; Mckee & Weinberger, 2013).

In our empirical analyses, we analyzed two waves of repeated assessments from the Netherlands Twin Register (Ligthart et al., 2019) to compare the CLPM and the IV-CLPM models, examining bidirectional causal influences between cigarette smoking status and alcohol use (drinks per week). Through this example, we demonstrate the etiological insights obtained by incorporating IVs into the CLPM, as well as the utility of using genetic variants (identified in genome-wide association studies) as IVs in large-scale panel studies with genetic data.

## Methods

### Instrumental variables regression

The SEM specification of the Instrumental Variables Regression (IVR) model (Maydeu-Olivares et al., 2019) is shown in Figure 1(B). In this model, the effect of *X* on *Y* can be estimated using the instrumental variable for *X* (*IVx*), even if *X* and *Y* are assessed simultaneously. Per this model, *X* completely mediates the effect of *IVx* on *Y*, such that *IVx* has no direct effect on *Y*. The IVR model also allows us to estimate the covariance between the residuals of *Y* and *X* (${\mathrm{Cov}}_{\mathrm{Exy}}$), in addition to the coefficient of the regression path from *X* to *Y* ($b_{YX}$). If the correlation between the residuals of *Y* and *X* is small, standard regression approaches may be appropriate for estimating the approximate causal effect of *X* on *Y*. By adding the instrumental variable *IVx* to the model, this assumption can be investigated empirically.

Formally, the instrumental variable, *IVx*, is required to satisfy three main assumptions (Labrecque & Swanson, 2018): (i) it is associated with *X* (“relevance”), (ii) it is not correlated with the residual variance of *Y*, given *X* (“exclusion restriction”), and (iii) it is independent of the (omitted) confounding variables (“exchangeability”). As shown by Maydeu-Olivares et al. (2020), under these assumptions, IVR can provide consistent estimates of the causal effect of *X* on *Y*, and it is robust to alternative sources of covariance between *X* and *Y*, including omitted confounding variables, reciprocal causation, reverse causation, and no causation. As is the case in any statistical model, inference of the causal estimates in IVR depends on the IV assumptions being satisfied. However, it may not be possible to assess some of these assumptions empirically (such as “exclusion restriction”), behooving researchers to rely on theoretical reasoning for the selection of appropriate IVs. In addition, sensitivity analyses may help to examine the robustness of the causal estimates to assumption violations.

### IV-CLPM

The proposed IV-CLPM model is shown in Figure 1(C). The model incorporates two IVs, *IVx* and *IVy*, into the traditional CLPM with two time-points, thus allowing for the IVR model to be applied cross-sectionally at each study wave. Adding IVs to the CLPM allows us to estimate three types of causal effects between *X* and *Y*. First, the IVR-estimated cross-sectional (i.e., proximal) effects at wave 1 ($b_{Y1X1}$ and $b_{X1Y1}$) reflect the causal process that unfolded *up to* the first assessment. Second, the lagged (i.e., distal) effects of $X_{1}$ on $Y_{2}$ ($b_{Y2X1}$) and of $Y_{1}$ on $X_{2}$ ($b_{X2Y1}$) represent the Granger-causal influences between *X* and *Y*, given the time interval between waves 1 and 2. Third, the IVR-estimated proximal effects at wave 2 ($b_{Y2X2}$ and $b_{X2Y2}$) reflect the causal influences that unfolded between waves 1 and 2 but were not captured by the distal effects. Thus, the wave-2 proximal effects represent *conditional* IVR estimates, controlling for the IVR estimates at wave 1 and the distal Granger effects. The terms “proximal” and “distal” underscore the temporal relationship between the predictor and the outcome for that estimate, indicating whether the outcome (e.g., $Y_{2}$) was assessed simultaneously ($b_{Y2X2}$) or on a subsequent occasion ($b_{Y2X1}$).

### Data generation

**Figure 2.**
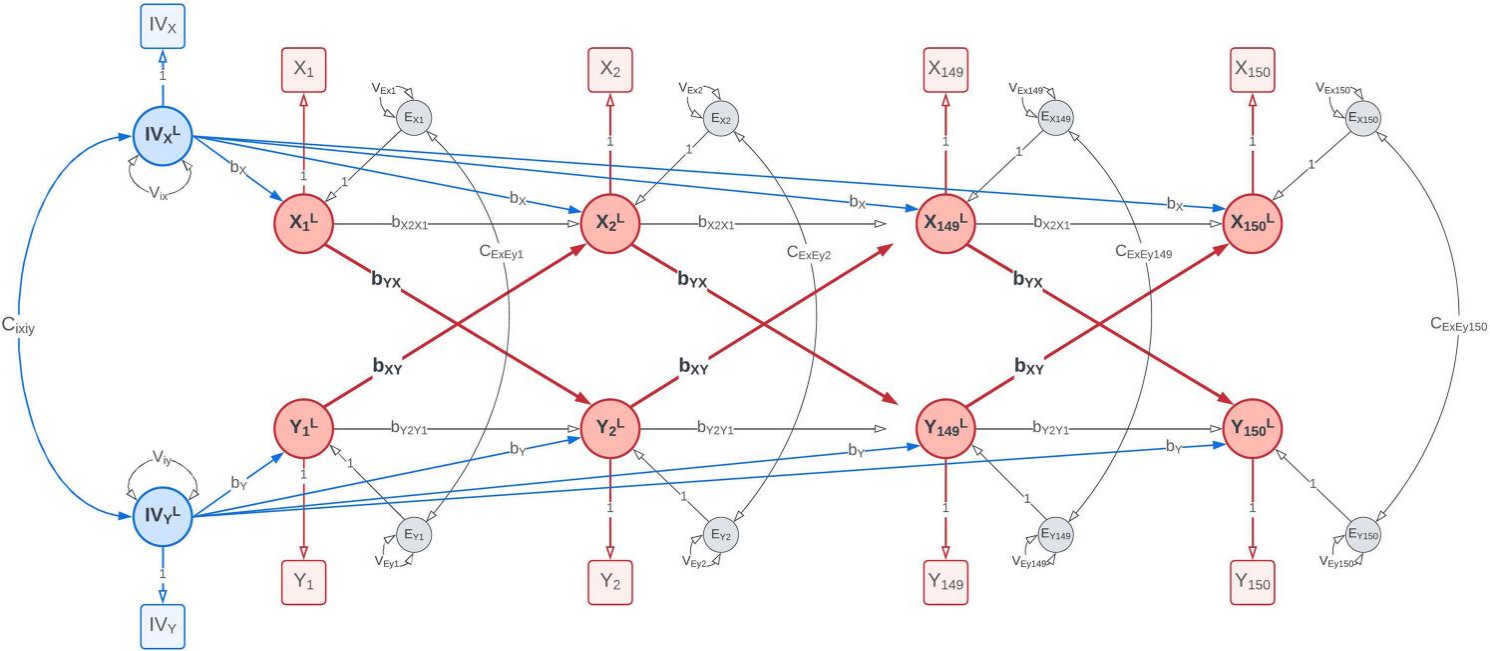
Data-generating model: The data-generating model with bidirectional first-order causal effects between *X* and *Y* ($b_{YX}$ and $b_{XY}$) simulated over 150 time points. The instrumental variable for *X*, *IVx*, has an unchanging direct effect on *X* ($b_{X}$) at every time point. Likewise, the instrumental variable for *Y*, *IVy*, directly affects *Y* ($b_{Y}$) at all time points. Squares/rectangles represent the observed variables, and circles represent latent variables (i.e., the variances in this model). To improve readability, the modeling of means is not shown in this figure. For a complete path diagram with means, please see Figure A2 in the Appendix A.

We simulated time-series data with reciprocal causal effects between two variables, *X* and *Y* (Figure 2), to compare our proposed IV-CLPM with the traditional CLPM. We included two IVs in the simulated data: *IVx* with a direct effect on *X*,   and *IVy* with a direct effect on *Y*, on every occasion. In the data-generating model, we only include lagged paths to represent the causal effects between *X* and *Y*. This specification is consistent with the expectation that, in the true causal process, the cause temporally precedes the outcome.

To generate the data, we calculated the expected covariance matrix with a selected set of parameter values, given T=150 time points, using the Reticular Action Model (RAM; McArdle & McDonald, 1984) formula for the covariance structure:

$$\Sigma =(I-A{)}^{-1}\;S\;(I-A{)}^{-1T},$$

where **Σ** is the expected covariance matrix. As mentioned in the introduction, the time scale of these 150 repeated measurements would depend on the variables studied, potentially varying from milliseconds to years. Given two variables (*X* and *Y*) on each of the *T* time points, plus two IVs (*IVx* and *IVy*), **Σ** is m×m, where m=2(1+T). The matrix A (m×m)  contains the bidirectional proximal and distal regression coefficients, the coefficient of the regression of *X* on *IVx*, and the coefficient of the regression of *Y* on *IVy*. The matrix S (m×m) contains the variances and covariance of *IVx* and *IVy*, the residual variances of *X* and *Y*, and the cross-sectional covariances of these residuals.

Given the m×m covariance matrix **Σ**, we simulated data with an arbitrary N=1000, using the mvrnorm() function in the *MASS* package (Venables & Ripley, 2002), with the option empirical = TRUE (i.e., exact data simulation; van der Sluis et al., 2008). This method ensures that the covariance matrix of the simulated data exactly equals **Σ** and that the true parameter values are recovered exactly upon fitting the true model to the data. In other words, the exact-data simulation approach is analogous to fitting a model to the exact population covariance matrix and means vector. Furthermore, this approach ensures that the likelihood-ratio test statistic obtained when a parameter is fixed to zero equals the non-centrality parameter of the non-central chi-square distribution, with no stochastic variation.

We simulated multiple datasets using different permutations of parameter levels for data generation, allowing us to examine the impact of population parameters on the changes in causal estimates across time intervals. These parameters included the first-order causal path from *X* to *Y*, $b_{YX}\in (0.1,\;0.2),$ the first-order causal path from *Y* to *X*, $b_{XY}\in (0.1,\;0.2),$ the first-order autoregressive path (AR1) of *X*, $b_{X2X1}\in (0.5,\;0.7),$ AR1 of *Y*, $b_{Y2Y1}\in (0.5,\;0.7),$ and the cross-sectional correlation between the residuals of *X* and *Y*, $r_{\mathrm{exy}}=C_{\mathrm{ExEyi}}/\sqrt{V_{\mathrm{Exi}}\times V_{\mathrm{Eyi}}}\;\in (0.1,\;0.3),$ set to be constant across all occasions. Both IVs were standardized to have a mean of 0 and a variance of 1, and had a correlation, $r_{IV}=r(\mathrm{IVx},\mathrm{IVy})=0.25.$ The direct effect of *IVx* on *X* on every occasion was set at $b_{X}=0.08,$ while the direct effect of *IVy* on *Y* was $b_{Y}=0.08.$

To examine the impact of time intervals on the causal estimates, we considered a stationary model. In a stationary time-series, the time index (i) has essentially no impact on the cross-sectional covariances ($\Sigma_{i})$ among the variables (Gagniuc, 2017; Ryabko, 2019). When parameters are estimated in such a system, they depend on the time interval, $\Delta T\;=T_{i}\;\text{-}\;T_{j}\;(i>j),$ but not on the particular time points $T_{i}$ and $T_{j}.$ To operationalize stationarity in this report, we consider it achieved when the elements of $\Sigma_{i}$ differ from those of $\Sigma_{i-1}$ by <0.0001.

The data-generating model used in this study is a Markovian process, i.e., the information at time point $T_{i}$ subsumes all past information and is sufficient to predict the information at the subsequent time point $T_{i+1}.$ The system is also time-homogeneous, i.e., the values of the causal paths do not change with the time index. When such a time-series is simulated with admissible parameter values over an extended number of time points, it usually meets the stationarity criteria beyond a certain time point. We established that the data-generating model achieved stationarity by T=70, given $b_{YX}=0.2,$ $b_{XY}=0.2,$ $b_{X2X1}=0.7,$ $b_{Y2Y1}=0.7,$ $r_{\mathrm{exy}}=0.1.$ When the AR1 ($b_{X2X1}\;$ and $b_{Y2Y1}$) parameters were smaller, the model reached stationarity at an earlier time point. We discarded data before T=100, thus ensuring stationarity. (See Appendix Table A1 for the correlations between variables over a range of time points beyond T=100.)

At stationarity, the effect size of an IV on its respective variable depended on the values of the other data-generating parameters. Thus, the $R^{2}$ for the regression of *X* on *IVx* in the stationary model ranged from 2.09% (given $b_{YX}=0.1,$ $b_{XY}=0.1,$ $b_{X2X1}=0.5,$ $b_{Y2Y1}=0.5,$ $r_{\mathrm{exy}}=0.3$) to 8.03% (given $b_{YX}=0.2,$ $b_{XY}=0.2,$ $b_{X2X1}=0.7,$ $b_{Y2Y1}=0.7,$ $r_{\mathrm{exy}}=0.1$). The regression of *Y* on *IVy* had an equivalent $R^{2},$ as similar parameter values were used for *X* and *Y*. These parameter levels were chosen to be consistent with modest-sized regressions of behavioral and psychiatric traits on exogenous IVs (e.g., polygenic scores), and with commonly observed AR1 parameter estimates.

**Figure A3.**
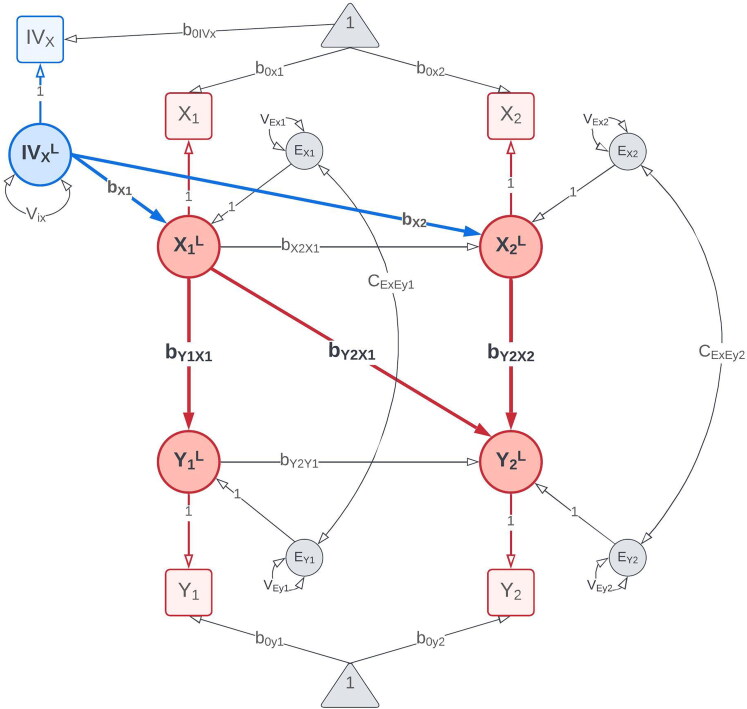
Unidirectional IV-CLPM. The model combines a unidirectional version of the two-wave Cross-Lagged Panel Model (CLPM) with Instrumental Variables Regression (IVR) applied cross-sectionally at each wave. In addition to the lagged (i.e., “distal”) effect of *X* on *Y* ($b_{Y2X1}$), the model utilizes IVR to estimate cross-sectional (i.e., “proximal”) effects at each wave: $b_{Y1X1}$ at wave 1, and $b_{Y2X2}$ at wave 2. The squares/rectangles represent the observed variables, and circles represent latent variables. Triangles represent constants used to model the variables’ mean levels.

We also considered a unidirectional version of the IV-CLPM, which uses the instrumental variable *IVx* to estimate the proximal and distal effects of *X* on *Y* (Appendix Figure A3). To test this model and compare it to the bidirectional version, we simulated a time-series with unidirectional causal effects of *X* on *Y* (i.e., with the effect of *Y* on *X* set to zero), given N=1000 and the following parameter settings: $b_{YX}=0.4,$ $b_{XY}=0,$ $b_{X2X1}=0.8,$ $b_{Y2Y2}=0.8,$ $r_{\mathrm{exy}}=0.3,$ $b_{X}=0.1,$ $b_{Y}=0.1,$ and $r_{IV}=0.$ The time-series reached stationarity by around T=50. To ensure stationarity for model-fitting, we discarded the data before T=100. At stationarity, the $R^{2}$ for the regression of *X* on *IVx* was 8.26%.

### Model fitting

**Figure A4.**
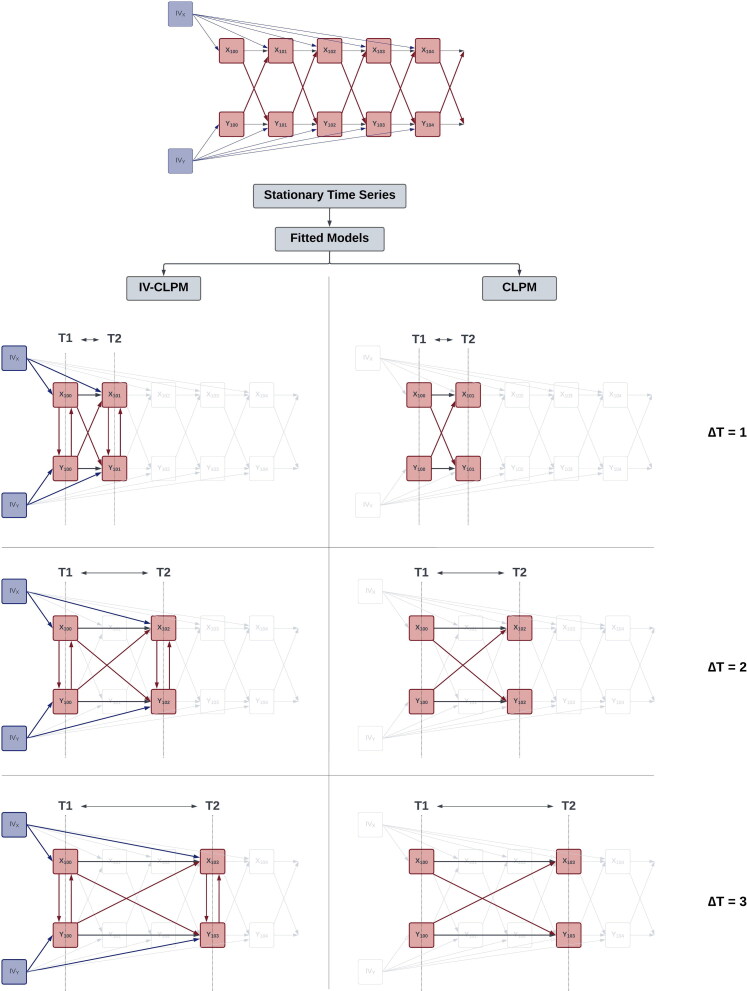
Schematic of the study design for examining the impact of time interval on the causal inference in the proposed IV-CLPM and the traditional CLPM models. A stationary time series was generated with two constructs (*X* and *Y*) and their respective instrumental variable (*IVx* and *IVy*) with bidirectional first-order lagged effects between *X* and *Y*. To this data, a series of two-wave IV-CLPM and CLPM models were fitted. Across the models, the first wave (T1) was fixed at an arbitrary time-point in the stationary time series, while the second wave (T2) was changed by an increment of one unit in every successive model. In so doing, the time interval (ΔT=T2−T1) in the fitted models was increased sequentially from 1 through 50. This figure depicts the models with ΔT=1, 2, and 3.

To each simulated dataset, we fitted a series of IV-CLPMs with varying time intervals. To do so, we set wave 1 at time point T=100 and wave 2 at T+ΔT, and we changed the ΔT sequentially from 1 to 50, thus increasing the time interval (Appendix Figure A4). Because the data-generating model is stationary, the 50 models fitted to a particular dataset differed only in the time interval between the two waves. This design allowed us to examine how the time interval between study waves influences the distal and proximal effects estimated in the IV-CLPM (Figure 1(B)). To compare the IV-CLPM with the traditional CLPM, we also fitted models without the IVs and the proximal effects to obtain distal effects in the standard CLPM (Figure 1(A)), using the same datasets. Further, the datasets simulated using different parameter values allowed us to examine the impact of population parameters on the causal estimates, given varying time intervals in a stationary model.

We tested the causal parameter estimates in both models using likelihood-ratio tests (LRTs; Wilks, 1938). The LRT statistic equals the difference between the −2× log-likelihood (−2lnL) of the freely estimated model and that of a nested, restricted model with one or more of the causal parameters fixed to zero. When using exact data simulation, the LRT statistic from fixing a parameter equals the non-centrality parameter (NCP) of non-central chi-square distribution, which can be used to calculate the statistical power to reject the null hypothesis (van der Sluis et al., 2008). Thus, larger NCP values indicate higher power to estimate the tested parameter.

In the traditional CLPM, bidirectional causation can be tested using a two-degrees-of-freedom (2df) LRT of the reciprocal distal effects between *X* and *Y*. If the 2df LRT is significant, follow-up 1df tests can be used to discern the distal effect in each direction of causality. By contrast, in the IV-CLPM, the causal influences can be first tested through a 6df LRT of all three types of bidirectional causal effects (across time, at wave 1, and at wave 2), providing an omnibus test of causal influences between *X* and *Y*, given the data from two study waves. A significant omnibus test can be followed up with a 3df joint LRT of the three causal effects in each direction, followed by separate 1df LRTs of each causal parameter.

To assess whether the IV-CLPM provides a better modeling approach than the CLPM at a particular time interval, we conducted a joint 2df LRT of (unidirectional) distal and wave-2 proximal effects in the former, and compared it with the CLPM’s 1df LRT of distal effect. Both these LRTs test the null hypotheses for the causal influences occurring between waves 1 and 2. So, the larger the difference between the NCPs of the two tests, the more substantial the benefit of fitting the IV-CLPM and estimating the wave-2 proximal effect.

We also fitted both the unidirectional and the bidirectional IV-CLPM to the dataset simulated with unidirectional causation. As before, we set wave 1 at T=100 and wave 2 at T+ΔT, with ΔT increasing sequentially from 1 to 50. We compared the NCPs in the two models to investigate whether fitting the unidirectional model increases the power to detect causation (vs. the bidirectional model) in data with unidirectional effects.

### Empirical application

We used both the CLPM and the IV-CLPM models to examine bidirectional causal influences between cigarette smoking status and alcohol use (drinks per week), using relevant genetic variants as IVs. For these analyses, we used data from the Netherlands Twin Register (NTR; Ligthart et al., 2019), which is a community-based longitudinal study of twins and their families, established at the Vrije Universiteit (VU) Amsterdam. The NTR study has been approved by the Central Ethics Committee on Research Involving Human Subjects of the VU Medical Center, Amsterdam (IRB codes 2008/244 and 2010/359). Informed consent was obtained from all study participants before data collection.

The NTR collects data on health, behaviors, personality, and lifestyle factors, along with biological samples, including DNA from whole blood and buccal tissue. The DNA samples have been genotyped on single-nucleotide polymorphism (SNP) microarrays. The human genome has ∼3.2 billion nucleotide base pairs (the smallest unit of DNA). A SNP is a genetic locus that is one base-pair long, where more than one type of nucleotide exists in the population; hence the term SNP (often pronounced “snip”). Tens of millions of SNPs have so far been identified in the global human population (Auton et al., 2015). SNP microarrays provide a scalable laboratory tool for measuring (i.e., “genotyping”) a subset of the common SNPs (typically around a million) that an individual carries (Wang et al., 1998). These genotyped SNPs can then be analyzed as markers to identify genetic loci associated with a trait of interest. Such a study design is known as a genome-wide association study or GWAS (Uffelmann et al., 2021). As explained below, the SNPs associated with a trait (or a weighted sum of such SNPs) can serve as potential IVs (provided other IV assumptions are satisfied).

#### Participants

In the current analyses, we included genotyped European-ancestry adult individuals with two waves of survey data collected three years apart: the Adult NTR (ANTR) Survey 8 (in 2009) and the ANTR Survey 10 (in 2012). (Henceforth, we refer to these two surveys as waves 1 and 2, respectively.) To avoid the clustering of study participants within families, we selected one individual per family for the current analyses. In these analyses, we included data from 4895 individuals, with 3983 and 3803 participants having non-missing observations at waves 1 and 2, respectively. This sample comprised 1745 males and 3150 females (self-reported gender, matched with biological sex inferred from the genotype). The age at wave 1 ranged from 15 to 94 years (Mean=43.95, S.D.=15.87 years), while wave 2 had an age range of 18 to 90 years (Mean=46.96, S.D.=16.71 years). Sex and age were used as covariates with fixed effects at each wave in both the CLPM and the IV-CLPM.

#### Measures

**Figure 3.**
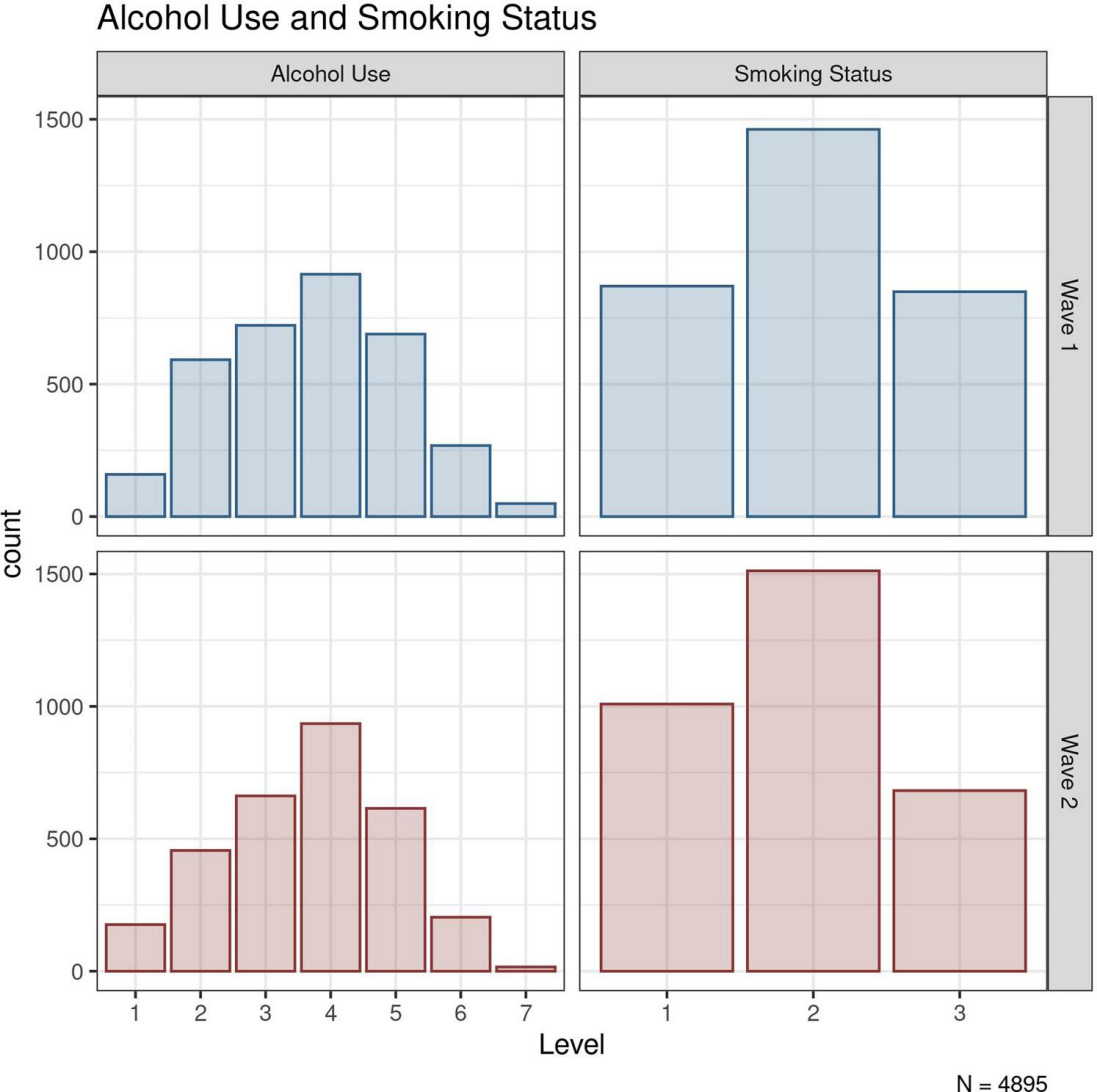
The univariate distributions of alcohol use and smoking status variables at wave 1 (Adult NTR survey 8) and wave 2 (Adult NTR survey 10) in the Netherlands Twin Register data used in our empirical example. Alcohol use was operationalized as the number of alcoholic drinks per week, with the seven levels corresponding to <1, 1–2, 3–5, 6–10, 11–20, 21–40, and >40 drinks per week, respectively. The cigarette smoking status variable was a categorical variable with three response options: 1 = “Never smoked regularly,” 2 = “Used to smoke but quit” (i.e., former smoking), and 3 = “Currently Smoking.”

Current cigarette smoking status and alcohol use were measured through self-reports. ***Smoking status*** assessment consisted of three response options: “*Never smoked regularly*,” “*Used to smoke but quit*” (i.e., former smoking), and “*Currently Smoking*.” We treated this measure as an ordinal variable with a normally distributed latent liability, under the *Liability Threshold Model* (Verhulst & Neale, 2021). We fixed the two thresholds (corresponding to the three levels) at −0.5 and 0.5, allowing us to freely estimate the mean and variance of the underlying liability scale at each wave (Mehta et al., 2004). ***Alcohol Use*** was operationalized as the number of alcoholic drinks consumed per week. The participants reported their current consumption of a variety of alcoholic drinks in a typical week, which was aggregated into a seven-point scale (<1, 1–2, 3–5, 6–10, 11–20, 21–40, and >40 drinks per week). We treated this variable as a continuous variable. Figure 3 shows the distribution of both traits at each wave (four variables in total). The numbers of non-missing observations on each variable and the pairwise subject overlap between variables are shown in Table 1.

**Table 1.** The number of observations of each variable and pairwise overlap between variables.

	Alcohol at T1	Alcohol at T2	Smoking at T1	Smoking at T2
Alcohol at T1	**3394**			
Alcohol at T2	2074	**3064**		
Smoking at T1	2582	1929	**3181**	
Smoking at T2	2123	2464	2336	**3203**

*Note.* The study sample comprised 4895 individuals, of whom 3983 and 3803 individuals provided data at waves 1 and 2, respectively. The number of observations per variable are listed along the diagonal (highlighted). The off-diagonal values indicate the number of observations of pairwise overlap between variables.

#### Genetic instrumental variables

We used a weighted sum of SNPs (i.e., a polygenic score, or PGS) associated with smoking status and drinks per week as their respective IV. A typical SNP involves two variant forms (called “alleles”) at a base-pair position in the population, say *A_1_* and *A_2_*. As humans have two sets of chromosomes (one from each biological parent), there are three possible allele combinations for a given SNP (except the SNPs on sex chromosomes) in an individual: *A_1_A_1_*, *A_1_A_2_*, or *A_2_A_2_*. The SNP can be coded according to the number of trait-increasing alleles (say, *A_2_*) as 0, 1, and 2, respectively. A PGS is then computed as a weighted linear combination of these values across all SNPs associated with a trait. The weights are based on the SNP-trait association effect sizes estimated through GWAS in an independent, ancestry-matched sample (Wray et al., 2021). An individual’s PGS for a trait reflects their genetic propensity for that trait, relative to the general population. The utility of PGSs as IVs has previously been demonstrated in other SEM-based extensions of IVR (Castro-de-Araujo et al., 2023; Minică et al., 2018) and applied empirically (e.g., de Vries et al., 2021; Lim et al., 2022; Oginni et al., 2023).

In general, the use of genetic variants as IVs is referred to as ***Mendelian Randomization*** (MR) analysis (Davey Smith & Ebrahim, 2003; Lawlor et al., 2008). Here, the term “Mendelian Randomization” refers to the random segregation and independent assortment of genetic loci during gamete formation in the parents. Consequently, the alternative alleles of a SNP can be assumed to be randomly distributed at the population level and, thus, be independent of potential environmental confounding (i.e., the “exchangeability” assumption). Thus, genetic IVs are interpretable as Bollen (2012)’s “randomization IVs.” Another advantage of genetic IVs is that one can safely assume that there is no reverse causation from a trait, such as smoking, to the associated SNPs.

In this study, we used the results from large-scale European-ancestry GWAS meta-analyses of “smoking initiation” and “drinks per week” (Saunders et al., 2022), excluding the NTR from the GWAS meta-analysis, to derive PGSs associated with the smoking and alcohol use measures in the NTR (i.e., the “relevance” assumption). The PGSs were calculated using *LDpred* v0.9 (Vilhjálmsson et al., 2015). Both PGSs were residualized for the SNP microarray platform and the first 10 genetic principal components, and then standardized to have a mean of zero and S.D. of one. As a PGS summarizes the effects of many SNPs, it has a much larger effect size than the individual SNPs, reducing the risk of weak-instrument bias in the IV-CLPM. In the NTR, the residualized PGS of smoking explained 2.2 and 2.4% of the variance in the smoking status at waves 1 and 2, respectively (controlling for age and sex). Likewise, the PGS of drinks per week had an incremental $R^{2}$ of 1.2 and 1.0% at waves 1 and 2, respectively. Supplemental Methods describe in greater detail the methods and procedures used for genotyping and quality control of the genetic data, genetic principal component analysis, and PRS calculation.

The two (residualized) PGSs, used as IVs in this example, showed a Pearson correlation of r=0.196 (95% confidence interval = 0.168, 0.222). This correlation between the PGSs arises due to covariance between the SNP-smoking and SNP-alcohol associations (in the respective GWAS), suggesting a genetic overlap between the two traits (called “pleiotropy”). This overlap of genetic signals can arise through multiple mechanisms. On the one hand, this could imply a direct causal effect of one trait on the other. For instance, if alcohol use has a causal effect on smoking, the SNPs that influence alcohol use will indirectly also influence smoking. Consequently, these SNPs will be shared between the two PGSs, albeit with different weights. This type of genetic overlap (called mediated or “vertical” pleiotropy) forms the essence of MR analyses (Richmond & Davey Smith, 2022). On the other hand, the genetic overlap between traits (and, in turn, a correlation between their PGSs) can arise if the SNPs influencing trait *X* also influence trait *Y* via a pathway that excludes trait *X* (called unmediated or “horizontal” pleiotropy) (Richmond & Davey Smith, 2022). These SNPs will also be shared between the two PGSs. For instance, in the current analyses of smoking and alcohol use, SNPs in the *BDNF* (brain-derived neurotrophic factor) gene, which influences drug reward mechanisms, could show horizontal pleiotropy and influence the two traits separately (Liu et al., 2019, p. 240).

Horizontal pleiotropy, if not modeled, is a threat to the validity of genetic IVs (and standard MR analyses), as it violates the “exclusion restriction” assumption. However, the use of PGSs in the proposed IV-CLPM is arguably more robust to horizontal pleiotropy than standard MR approaches, as it allows for paths (or chains of paths) to connect the PGS of one trait with the opposite trait, independent of a direct causal effect between the two traits. Specifically, the model accounts for the covariance between the two PGSs, $C_{\mathrm{ixiy}}=\mathrm{cov}({IV}_{x},{IV}_{y})$ (Figure 1(C)). For instance, the PGS of smoking (${IV}_{x}$) is thus allowed to covary with alcohol use at wave 1 ($Y_{1}$) through the PGS of alcohol use (${IV}_{y}$), i.e., $C_{\mathrm{ixiy}}\times b_{y1},$ independent of smoking ($X_{1}$). In other words, the direct regression paths between the traits accommodate vertical pleiotropy (given true causal effects between the traits), while the covariance between PGSs potentially accommodates genetic confounding or horizontal pleiotropy. This use of PGSs as correlated IVs in a bidirectional IVR model, averting (to some extent) potential violations of the “exclusion restriction” assumption, is consistent with the model proposed in Castro-de-Araujo et al. (2023).

#### Model fitting

We fitted both the CLPM and the IV-CLPM models, controlling for sex and age at each wave. In both analyses, we began with the full model estimating bidirectional causal effects and then tested more restricted models to arrive at the most parsimonious model. As outlined above (in the case of simulated data), we constrained the relevant causal paths to zero and then compared the nested models using the LRT. In addition, we looked at the Akaike information criterion (AIC; Akaike, 1974). The AIC adjusts a model’s likelihood by including a penalty for the number of parameters estimated in the model, thus balancing model fit and model complexity (Vrieze, 2012). When comparing multiple models, we selected the model with the lowest AIC as the best-fitting, most parsimonious model.

All statistical analyses were performed using the *OpenMx* package (version 2.20.6; Neale et al., 2016) in the R statistical programming environment (version 4.1.2; R Core Team, 2021). We verified that all models were locally identified (Hunter et al., 2021). In the empirical example, we conducted statistical tests (LRTs) with an alpha of 0.05.

## Results

### Impact of time interval on causal inference in CLPM and IV-CLPM

#### Causal estimates

**Figure 4.**
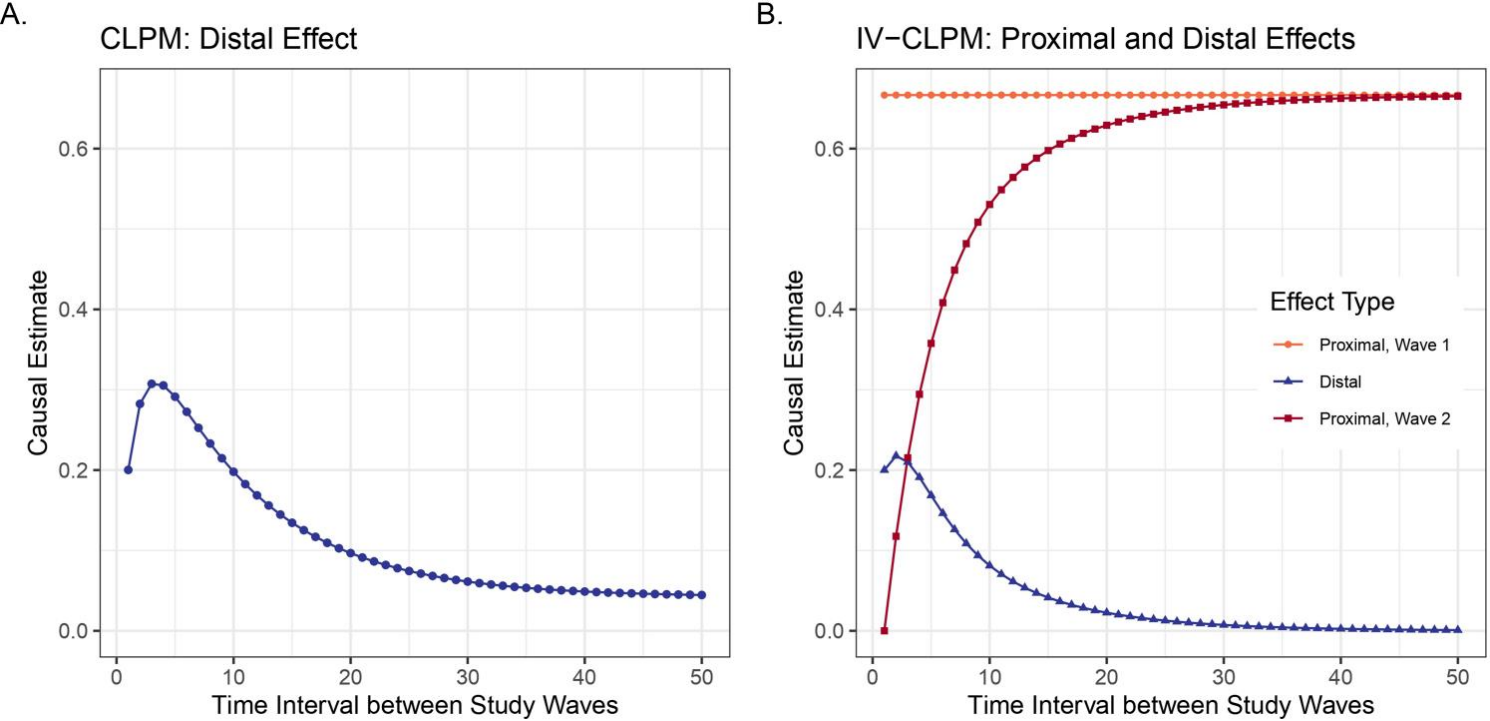
Impact of the time interval on (A) the distal effect in CLPM and (B) the distal and the two proximal effects in the IV-CLPM, with both models fitted to the data generated using the model in Figure 2. In the CLPM (A), the distal effect estimated increases briefly and then decreases asymptotically with increasing time intervals, varying from around 0.3 at an interval of 4 units to <0.05 at intervals longer than 30 units. In the IV-CLPM (B), the distal effect follows a pattern like that in the CLPM, but while the distal effect decays at longer intervals, the proximal effects can help estimate the causal effects. The plots illustrate the estimates for the effect of *X* on *Y*, given the first-order causal effect of *X* on *Y* = 0.2, the effect of *Y* on *X* = 0.2, first-order autoregression (AR1) of *X* = 0.7, AR1 of *Y* = 0.7, and the correlation between the residuals of *X* and *Y* = 0.3.

**Figure 5.**
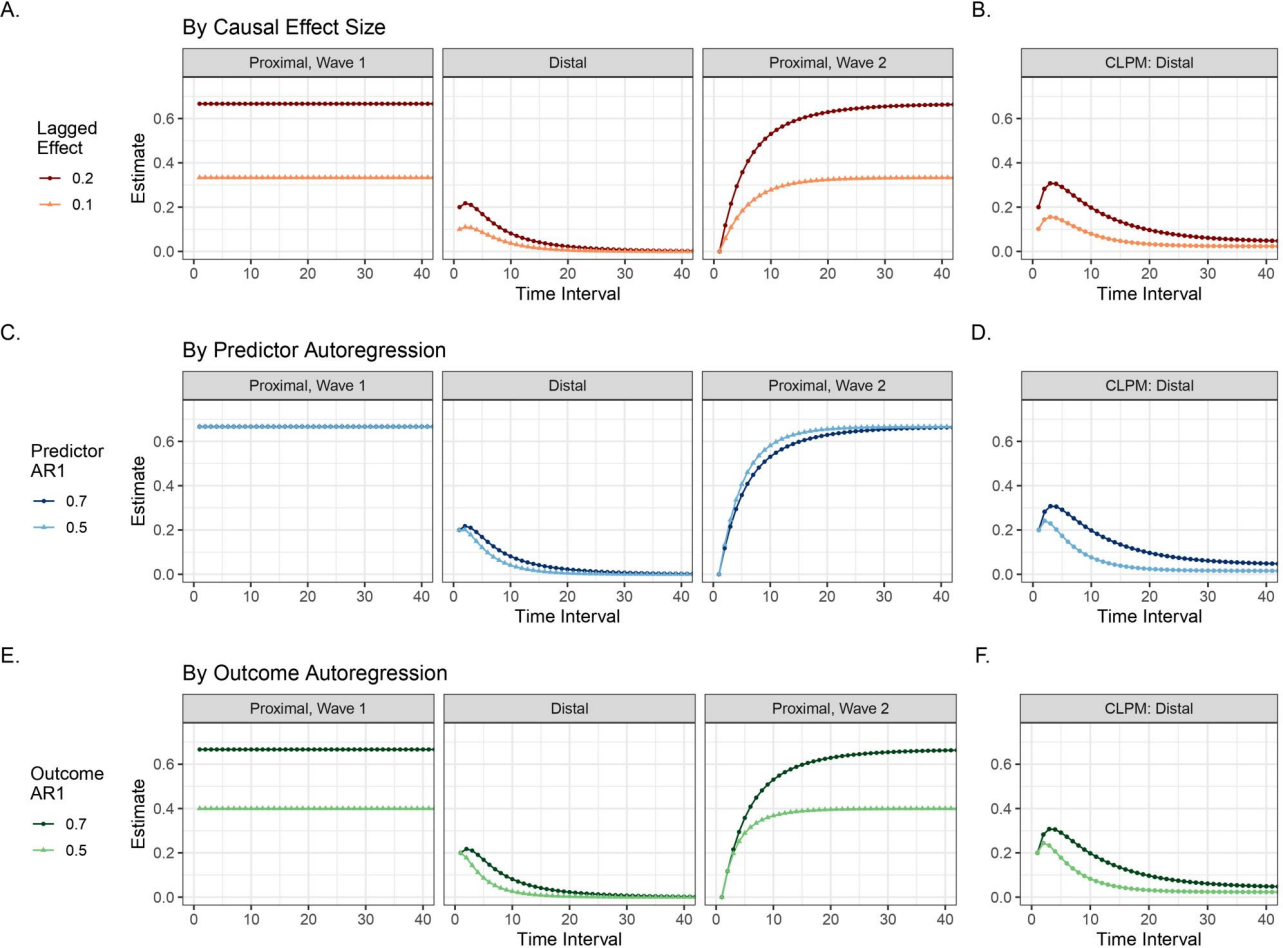
The estimated causal effect of *X* on *Y* in the IV-CLPM (Figure 1(C)) and the CLPM (Figure 1(A)) at varying time intervals between study waves, given different levels of the first-order (i.e., at ΔT=1) causal effect of *X* on *Y* (A,B), first-order autoregression (AR1) of predictor *X* (C,D), and AR1 of outcome *Y* (E,F) in the data-generating model (Figure 2). For ease of display, the time intervals are shown up to 40 units, by which point all parameter estimates are close to their asymptotes. (A,B) The first-order causal effect does not impact the rate at which the distal effect decays with increasing time intervals in either model. However, a larger first-order causal effect size does lead to larger causal estimates in both models (as expected). Note, though, that the distal effect in the IV-CLPM approaches zero at longer intervals, regardless of the first-order causal effect size. (C,D) A larger AR1 parameter (i.e., greater stability over time) of the predictor variable leads to a larger distal effect in the CLPM across time intervals, as well as slower decay of the distal effect with increasing intervals. On the contrary, the degree of AR1 in the predictor has minimal impact on the causal estimates in the IV-CLPM. Note that in panel C, the two curves for the Proximal Effect at Wave 1 have fully overlapped, so only one of the two curves is visible. (E,F) The AR1 parameter of the outcome variable impacts the causal estimates in both models: a higher level of outcome AR1 leads to larger causal estimates and slower decay of the distal effect in both models.

**Figure 6.**
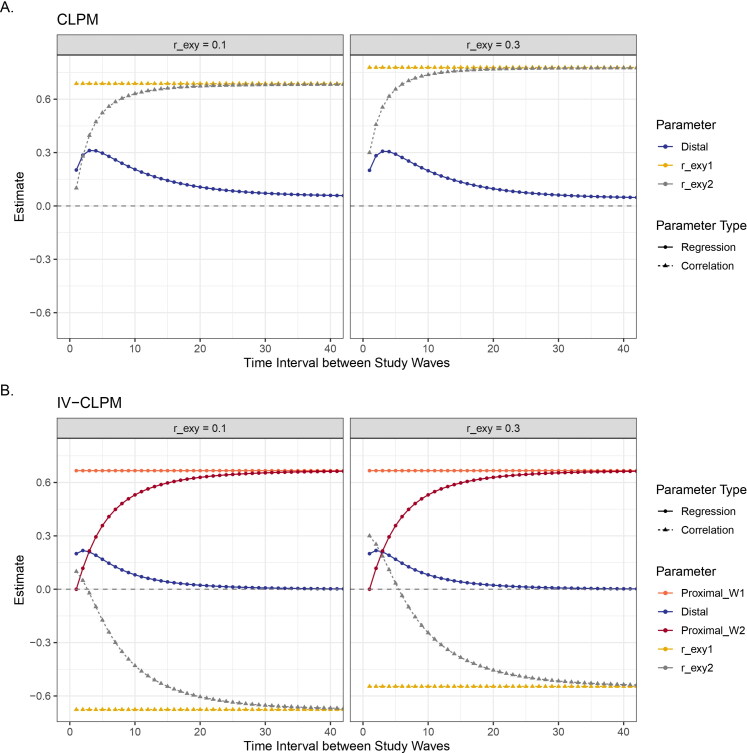
Variation in the causal estimates (effect of *X* on *Y*) and the cross-sectional correlation between the residuals of *X* and *Y* in (A) the CLPM and (B) the IV-CLPM, at varying levels of correlation between the residuals in the data (*r_exy*). The residual correlation in the data has a negligible impact on the causal estimates in either model, but affects the correlation of the residuals in the model, *r_exy1* and *r_exy2*. Given stationarity, the correlation of the residuals also varies with changes in the causal estimates, which, in turn, depend on the time interval between study waves. For ease of display, the time intervals are shown up to 40 units, by which point all parameter estimates are close to their asymptotes.

The distal effects in the traditional CLPM (Figure 4(A)) increase briefly as the time interval between study waves (ΔT) initially increases, and then gradually decreases to reach an asymptote close to zero at extended intervals. Consequently, in the example shown in Figure 4(A), the lagged effect would vary from around 0.3 at ΔT=5 to <0.05 at ΔT>30. The peak estimates during the initial rise and the estimates at longer time intervals are higher with a larger first-order causal effect size and stronger autocorrelations of the two variables (Figures 5(B,D,F)). Moreover, with stronger autocorrelation of either variable, the distal effects take longer to decay. The cross-sectional correlation between the residuals of the two variables does not affect the estimated distal effects in the CLPM (Figure 6).

The three causal effect types in the IV-CLPM show distinct patterns based on the time interval between study waves. The proximal effects at wave 1 do not vary with the time interval (Figure 4(B)). These estimates capture the causal influences that occurred *before* the first measurement occasion (i.e., wave 1 in the model), and so remain unchanged as the time point of wave 1 is unchanged. Distal effects in the IV-CLPM follow trends similar to those observed in the traditional CLPM: there is a brief initial increase in the distal effect size, followed by a steady decline across increasing time intervals. However, compared to the CLPM, the peak of the distal effect in the IV-CLPM at short intervals is attenuated. Also, in the IV-CLPM, the distal effect at longer time intervals approaches zero regardless of the first-order causal effect size and the autocorrelations of the two variables. Lastly, the proximal effects at wave 2 increase gradually as the time interval increases, reaching an asymptote that approaches the proximal effect at wave 1 (given stationarity). As expected, a larger first-order causal effect size and stronger autocorrelation of the outcome (Figures 5(A,E)) lead to higher estimates for all three effects (except for the distal effect at longer intervals, which invariably approaches zero). Unlike the CLPM, the autocorrelation of the predictor variable has little impact on the causal estimates in the IV-CLPM (Figure 5(C)).

#### Likelihood-ratio tests

**Figure 7.**
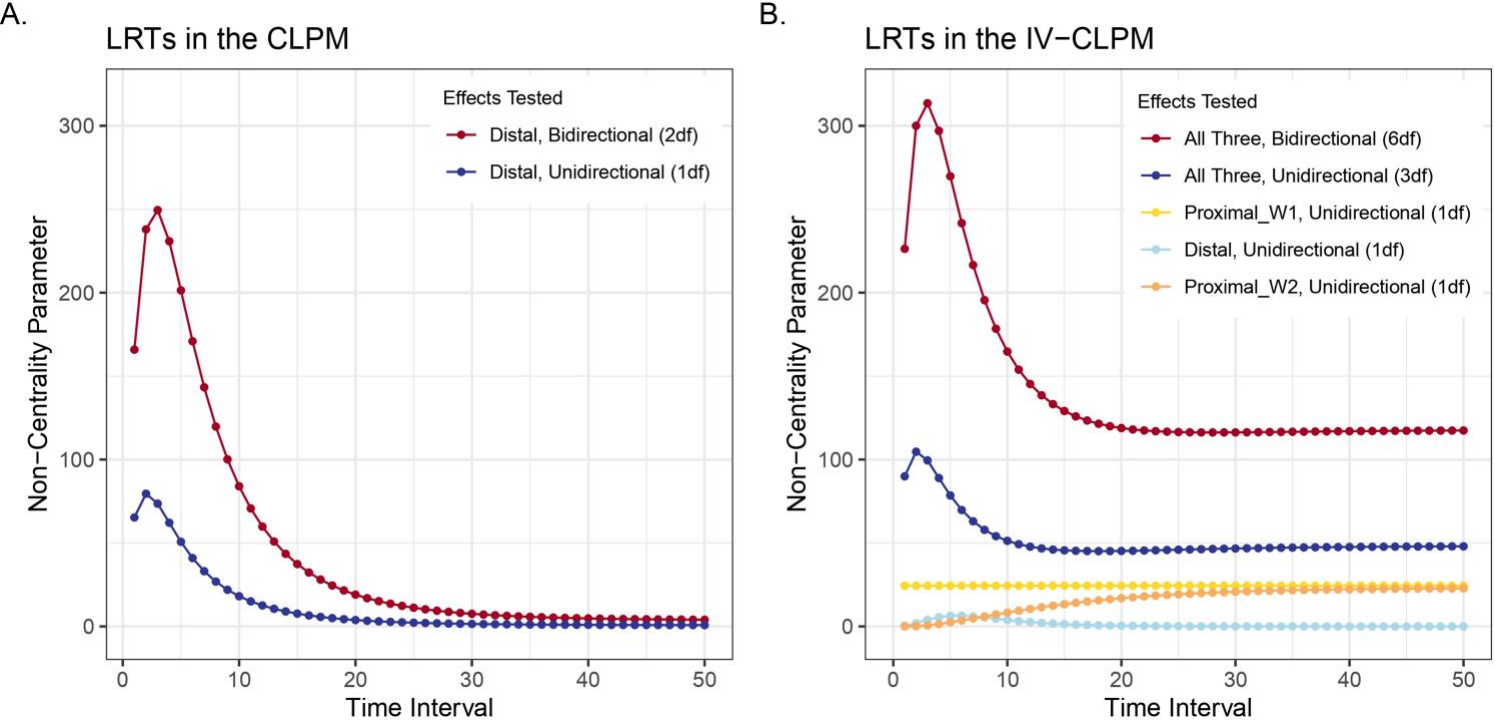
Likelihood-ratio tests (LRTs) of the causal estimates in the traditional CLPM and the IV-CLPM at varying time intervals between study waves. (A) CLPM: The non-centrality parameter (NCP) obtained from a 2-degrees-of-freedom (2df) test of bidirectional distal effects, and that obtained from a 1df LRT of a unidirectional distal effect (shown here is *X* to *Y*). (B) IV-CLPM: The NCP from a 6df omnibus test of bidirectional causal estimates of three types: the proximal effect at wave 1 (Proximal_W1), the distal effect, and the proximal effect at wave 2 (Proximal_W2). A significant omnibus test is followed up with a 3df LRT of the three causal effects in each direction, and, finally, the 1df LRTs of the three causal effects separately (in each direction of causation). The NCPs were obtained by fixing to zero the parameters of interest in models fitted to data with N=1000, $b_{YX}=0.2,$ $b_{XY}=0.$2, $b_{X2X1}=0.7,$ $b_{Y2Y1}=0.7,$ and $r_{\mathrm{exy}}=0.3.$

With increasing time intervals in the traditional CLPM, the 2df LRT of bidirectional distal effects and the 1df LRTs of the distal effect in each direction follow the patterns seen in the causal estimates (Figure 7(A)). The NCP obtained from these LRTs trends toward zero at longer time intervals. Therefore, at extended intervals, the small distal effects in the CLPM are unlikely to be detected.

In the IV-CLPM, the NCP obtained from an omnibus 6df LRT of bidirectional causation shows a similar overall pattern across increasing time intervals, first briefly increasing and then gradually decreasing toward an asymptote (Figure 7(B)). However, compared to the traditional CLPM’s 2df NCP (of the two distal effects), the asymptote reached by the IV-CLPM’s 6df NCP is substantially higher. Similarly, in each direction of causation, the 3-df NCP of the three causal estimates in the IV-CLPM is considerably larger than the CLPM’s 1df NCP of the distal effect. As the IV-CLPM has opportunities to resolve the causal process into three effect types, the power to detect these effects separately (i.e., through the 1df tests in each direction of causation) is necessarily lower than their joint 3df LRT.

### Choosing between CLPM and IV-CLPM

**Figure 8.**
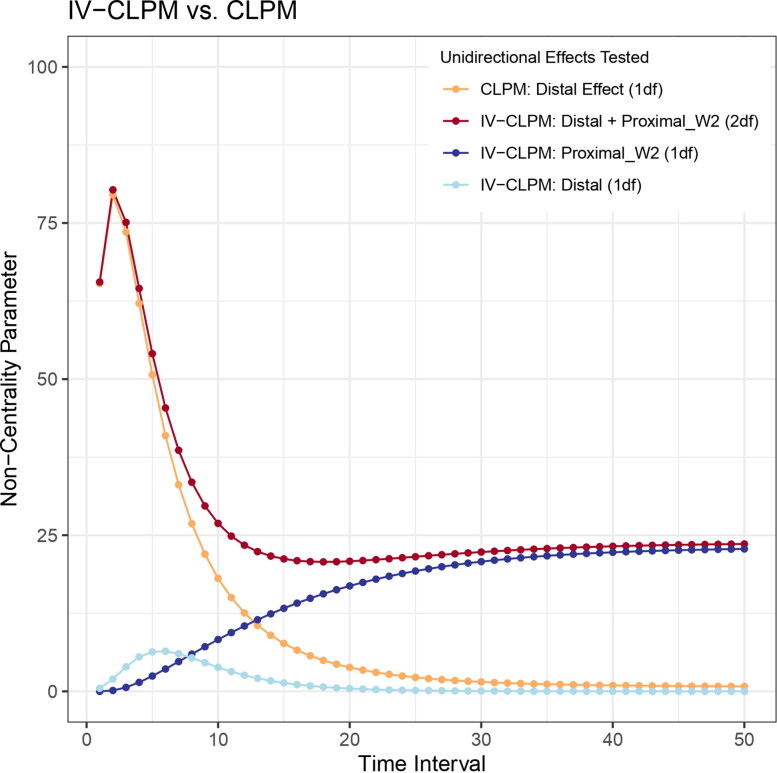
Comparison of the IV-CLPM’s joint test of (unidirectional) distal and wave-2 proximal effects with its 1df LRT of wave-2 proximal effect, as well as with the CLPM’s 1df LRT of distal effect. For reference, the IV-CLPM’s 1df LRT of distal effect is also shown. Comparing the 2df LRT statistic (dark red) with the 1df LRT of wave-2 proximal effect (dark blue) in the IV-CLPM can help gauge whether a particular time interval would be appropriate for fitting the traditional CLPM. The non-centrality parameters were obtained by fixing to zero the parameters of interest in models fitted to data with N=1000, $b_{YX}=0.2,$ $b_{XY}=0.$2, $b_{X2X1}=0.7,$ $b_{Y2Y1}=0.7,$ and $r_{\mathrm{exy}}=0.3.$

At shorter time intervals, the power to estimate the distal effect in the CLPM is higher than that for estimating the three causal effects separately in the IV-CLPM. Here, “short” is a relative term indicating that, for a particular causal process, the time interval between two repeated assessments is short enough for a lagged Granger effect to adequately capture most of the causal influences. In such a case, the traditional CLPM is likely well-suited for estimating lagged effects, although one cannot know so beforehand. Therefore, it would be desirable to gauge whether the time interval in a study is appropriate for fitting the traditional CLPM (estimating only the distal effects) *vs.* the IV-CLPM (estimating both proximal and distal effects). To do so, we first compare the IV-CLPM’s 2df LRT of (unidirectional) distal and wave-2 proximal effects to the CLPM’s 1df LRT of the distal effect (Figure 8). When the time interval between study waves is reasonably short, the 1df NCP from the CLPM is closely approximated by the 2df NCP in the IV-CLPM’s joint test of distal and wave-2 proximal effects. As the time interval increases, the difference between the two NCPs widens, suggesting an increasing benefit of fitting the IV-CLPM and resolving the causal influences into distal and proximal effects.

To evaluate whether the IV-CLPM would be a better modeling approach than the CLPM at a particular time interval, we can compare the 1df NCP of wave-2 proximal effect to the 2df NCP of distal and wave-2 proximal effects (in each direction of causation). If the former is much smaller than the latter, it would suggest that the time interval between study waves is reasonable for fitting the CLPM with this pair of variables. Also, the IV-CLPM will likely have relatively low power to estimate the distal and proximal effects separately. In this case, it would be prudent to test whether the wave-2 proximal effect can be constrained to be zero, such that the interpretation of the IV-CLPM’s distal effect is equivalent to the CLPM’s distal effect. The new distal effect in the IV-CLPM will then capture the causal influences that unfolded between waves 1 and 2. This proposed workflow would allow one to assess empirically whether the time interval is indeed appropriate for fitting the traditional CLPM and, if so, modify the IV-CLPM to estimate the distal effect one would obtain from the CLPM.

On the other hand, if the 1df NCP of wave-2 proximal effect (e.g., from *X* to *Y*) approximates the joint 2df NCP of distal and wave-2 proximal effects, this would indicate that (1) the study waves are too far apart to meaningfully detect Granger causal effects, even though *X* likely has a causal effect on *Y*; and (2) the proximal effects at wave 2 approximate the causal influences occurring between waves 1 and 2. At such extended time intervals, the benefit of using the IV-CLPM over traditional CLPM is evident, as the former retains the power to detect causation through proximal effects and joint tests, while the power to detect causality in the CLPM evaporates.

### A note on the correlation between the residuals

In the traditional CLPM, the cross-sectional correlation between the residuals of *X* and *Y* at wave 2, $r_{\mathrm{exy}2}=C_{\mathrm{ExEy}2}/\sqrt{V_{Ex2}\times V_{Ey2}},$ captures all sources of covariance besides the Granger causal effects. That is, the correlation subsumes the covariance arising due to the causal effects between *X* and *Y* that were not accounted for by the distal Granger effects. Therefore, with increasing time intervals in a stationary model, the distal effects decrease, and the correlation between the residuals increases (Figure 6(A)).

In the IV-CLPM, the cross-sectional covariance between *X* and *Y* at wave 1 is accounted for by the wave-1 proximal effects, the covariance path between $X_{1}$ and $Y_{1},$ as well as the covariance between the two IVs. As the proximal effects represent the *cumulative* causal influences accrued up to that point, these effects can be larger than the observed cross-sectional covariance between $X_{1}$ and $Y_{1}.$ Consequently, the correlation of their residual variances at wave 1 in the IV-CLPM ($r_{\mathrm{exy}1}=C_{\mathrm{ExEy}1}/\sqrt{V_{Ex1}\times V_{Ey1}}$) may be negative, even if the reciprocal causal effects and the correlation of the residuals in the population (i.e., the simulated data in this paper) are all positive (Figure 6(B)). Furthermore, at wave 2, the distal effects, the wave-2 proximal effects, and the covariance between the residual variances of $X_{2}$ and $Y_{2}$ ($C_{X2Y2}$), provide additional paths contributing to the total covariance between $X_{2}$ and $Y_{2}.$ As the distal and the wave-2 proximal effects change at different time intervals, the correlation of wave-2 residuals ($r_{\mathrm{exy}2}=C_{\mathrm{ExEy}2}/\sqrt{V_{Ex2}\times V_{Ey2}}$) can be positive, negative, or zero (given stationarity). Therefore, the correlation between the residuals in the IV-CLPM may be difficult to interpret substantively.

### Bidirectional vs. unidirectional IV-CLPM in data with unidirectional causation

**Figure A5.**
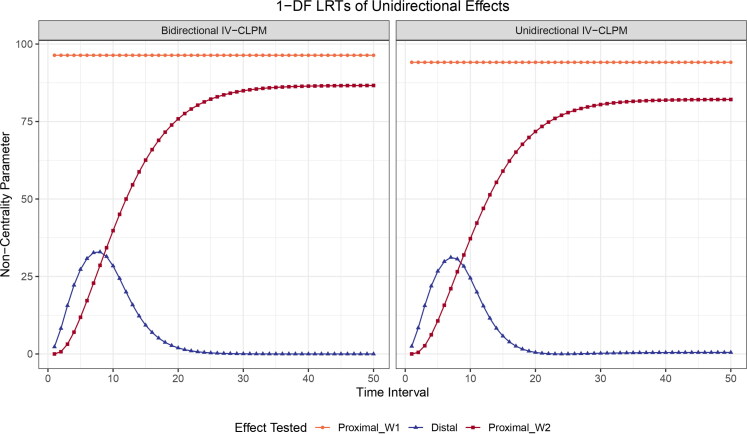
Likelihood-ratio tests (LRTs) of the effects of *X* on *Y* in the bidirectional (left) and unidirectional (right) versions of the IV-CLPM, given data with unidirectional effects of *X* on *Y*. The non-centrality parameters were obtained by fixing to zero the causal parameters in models fitted to data with N=1000, $b_{YX}=0.4,$ $b_{XY}=0,$ $b_{X2X1}=0.8,$ $b_{Y2Y1}=0.8,$ and $r_{\mathrm{exy}}=0.3.$

We obtained the NCPs from the 1df LRTs of the three causal effects of *X* on *Y* in both the unidirectional and the standard bidirectional IV-CLPM fitted to the dataset with unidirectional causation (*X* causing *Y*). For all three effect types (wave-1 proximal, distal, and wave-2 proximal effects), the NCPs obtained from both models followed similar trends with increasing time intervals, consistent with the pattern seen using data with bidirectional causation (Appendix Figure A5). However, the NCPs in the unidirectional model were slightly smaller than the equivalent statistics in the bidirectional model. Therefore, even though the unidirectional IV-CLPM is the more parsimonious model, given data with unidirectional causation, it does not provide any power advantage over the bidirectional model.

### Empirical examination of smoking and alcohol use

#### CLPM

**Figure 9.**
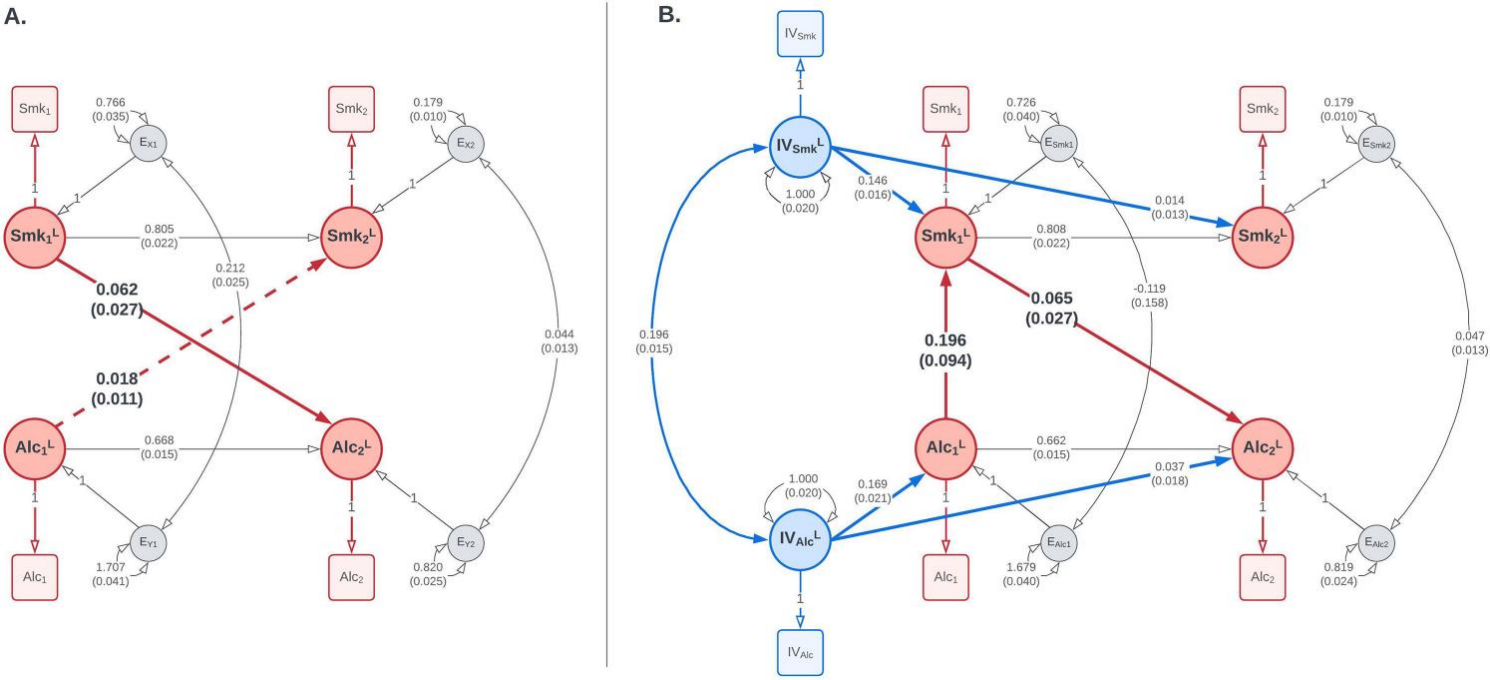
Results of (A) the CLPM and (B) the best-fitting IV-CLPM examining bidirectional causal effects between smoking status (Smk) and alcoholic drinks per week (Alc), assessed three years apart. The paths have been labeled with the point estimate and its standard error (in parentheses). The dashed path in the CLPM indicates a non-significant causal estimate. The CLPM suggests a likely unidirectional causal process, with a significant effect of smoking on alcohol use, but not *vice versa*. On the contrary, the IV-CLPM suggests a more complex bidirectional causation, with a significant proximal effect of alcohol use on smoking, which, in turn, has a reciprocal distal effect on alcohol use. In both path diagrams, squares/rectangles represent the observed variables, and circles represent latent variables. To improve figure readability, means and covariates are not shown in this figure. For a complete path diagrams with means and covariates, please see Figure A6 in the Appendix A.

Examining the bidirectional causal influences between smoking status and alcohol use (drinks per week) with the CLPM (Figure 9(A)), an omnibus LRT of both causal paths (Table 2) was statistically significant (NCP=8.29, df=2, p=0.016). Testing the two causal paths separately, we found a significant lagged effect of smoking on alcohol use (NCP=5.21, df=1, p=0.022), while the reverse lagged effect of alcohol use on smoking was non-significant (NCP=2.75, df=1, p=0.097). However, a restricted model with the effect of alcohol use on smoking set to zero did marginally increase the model AIC (ΔAIC=0.29), suggesting a slightly less parsimonious model fit. Thus, the estimates from the CLPM indicated a significant effect of smoking status on alcohol use ($b_{Y2X1}=0.062,$ S.E.=0.027), but a non-significant reverse effect ($b_{X2Y1}=0.018,$ S.E.=0.011), given a time interval of three years. Table 3 shows all the parameter estimates from the full CLPM model.

**Table 2.** Model fit statistics and likelihood ratio rests in models examining causal influences between smoking status and alcohol use.

Base model	Comparison model	# Estimated parameters	−2ln*L*	*df*	AIC	LRT		
						NCP	△*df*	*p*
I. CLPM								
Full CLPM		18	31,699.08	12,828	31,735.08			
Full CLPM	No bidirectional lagged effects	16	31,707.37	12,830	31,739.37	8.29	2	0.0159
Full CLPM	No Smoking → Alcohol lagged effect	17	31,704.29	12,829	31,738.29	5.21	1	0.0225
Full CLPM	No Alcohol → Smoking lagged effect	17	31,701.83	12,829	31,735.83	2.75	1	0.0971
II. IV-CLPM								
Global tests								
Full IV-CLPM		31	59,109.02	22,605	59,171.02			
Full IV-CLPM	No bidirectional causal effects	25	59,123.10	22,611	59,173.10	14.08	6	0.0288
Full IV-CLPM	No Smoking → Alcohol causal effects	28	59,115.69	22,608	59,171.69	6.67	3	0.0833
Full IV-CLPM	No Alcohol → Smoking causal effects	28	59,115.97	22,608	59,171.97	6.94	3	0.0738
Smoking → Alcohol								
Full IV-CLPM	No proximal effects at waves 1 and 2	29	59,110.24	22,607	59,168.24	1.21	2	0.5451
Full IV-CLPM	No distal + wave-2 proximal effects	29	59,114.39	22,607	59,172.39	5.37	2	0.0683
Alcohol → Smoking								
Full IV-CLPM	No proximal effects at waves 1 and 2	29	59,113.10	22,607	59,171.10	4.07	2	0.1305
Full IV-CLPM	No distal + wave-2 proximal effects	29	59,111.70	22,607	59,169.70	2.67	2	0.2626
Best-fitting IV-CLPM (Smoking → Alcohol distal effect, plus Alcohol → Smoking proximal effect at wave 1)								
Full IV-CLPM	Best-fit IV-CLPM	27	59,112.89	22,609	59,166.89	3.86	4	0.4247
Best-fit IV-CLPM	No Smoking → Alcohol distal effect	26	59,118.65	22,610	59,170.65	5.77	1	0.0163
Best-fit IV-CLPM	No Alcohol → Smoking proximal effect	26	59,117.28	22,610	59,169.28	4.39	1	0.0362

−2ln*L*: −2 × log-likelihood of the model; *df*: degrees of freedom; AIC: Akaike information criterion; LRT: likelihood ratio test; NCP: non-centrality parameter = △−2ln*L*.

**Table 3.** Parameter estimates in the full CLPM applied to smoking status and alcohol use.

Parameter	Description	Estimate	*SE*	Wald’s *T*	*p*-Value
Regression paths					
bx2x1	*Smoking (Smk) at T1 → Smk at T2*	0.805	0.022	36.262	0.0000
by2x1	***Smk at T1 → Alcohol (Alc) at T2***	**0.062**	**0.027**	**2.284**	**0.0223**
bx2y1	***Alc at T1 → Smk at T2***	**0.018**	**0.011**	**1.661**	**0.0967**
by2y1	*Alc at T1 → Alc at T2*	0.668	0.015	44.592	0.0000
(Co)variances					
Vx1	*Residual variance of Smk at T1*	0.766	0.035	22.111	0.0000
Cx1y1	*Covariance of residuals of Smk and Alc at T1*	0.212	0.025	8.636	0.0000
Vy1	*Residual variance of Alc at T1*	1.707	0.041	41.985	0.0000
Vx2	*Residual variance of Smk at T2*	0.179	0.010	18.774	0.0000
Cx2y2	*Covariance of residuals of Smk and Alc at T2*	0.044	0.013	3.367	0.0008
Vy2	*Residual variance of Alc at T2*	0.820	0.025	33.453	0.0000
Covariates					
b_smk_age	*Age → Smk*	0.007	0.001	7.969	0.0000
b_alc_age	*Age → Alc*	0.009	0.001	8.024	0.0000
b_smk_female	*Female sex → Smk*	−0.086	0.030	−2.897	0.0038
b_age_female	*Female sex → Alc*	−0.807	0.039	−20.716	0.0000
Intercepts					
b0_smk1	*Intercept of Smk at T1*	−0.341	0.050	−6.819	0.0000
b0_alc1	*Intercept of Alc at T1*	3.814	0.062	61.430	0.0000
b0_smk2	*Intercept of Smk at T2*	−0.440	0.053	−8.350	0.0000
b0_alc2	*Intercept of Alc at T1*	3.576	0.066	54.242	0.0000

*Note.* Rows shown in bold indicate estimates of the causal paths between smoking status (latent liability scale of an ordinal variable with levels: never–former–current smoking) and alcohol use (drinks per week).

#### IV-CLPM

Table 2 shows that an omnibus LRT of all six causal paths in the IV-CLPM model (three in either direction of causation) was statistically significant (NCP=14.08, df=6, p=0.029). When testing the two directions of causation separately, the joint LRT of the three causal paths was statistically non-significant: for smoking → alcohol (NCP=6.67, df=3, p=0.083), and for alcohol → smoking (NCP=6.94, df=3, p=0.074). However, in both cases, the effect sizes are plausible, and fixing the causal paths to zero worsened the AIC (ΔAIC=0.67 and ΔAIC=0.95, respectively).

We did two additional joint LRTs in either direction of causation. First, we tested both proximal effects jointly (i.e., the causal paths added in the IV-CLPM, beyond the causal effect estimated in the CLPM). Second, we jointly tested the distal effect and the wave-2 proximal effect (i.e., the causal influences between waves 1 and 2). Based on the joint LRTs and the AICs of the restricted models testing the causal paths from *smoking status to alcohol use* (Table 2), the best-fitting model (with the lowest AIC) was the one with both proximal effects constrained to zero (i.e., with only a distal effect of smoking on alcohol use). On the other hand, among the models testing the paths from *alcohol use to smoking status*, the best-fitting model (with the lowest AIC) had both the distal effect and the wave-2 proximal effect constrained to zero (i.e., with only a proximal effect of alcohol use on smoking status at wave 1).

Based on these LRTs, we retained a restricted IV-CLPM model (Figure 9(B)) with a distal effect of smoking status on alcohol use ($b_{Y2X1}=0.065,$ S.E.=0.027), and a reverse proximal effect of alcohol use on smoking status at wave 1 ($b_{X1Y1}=0.196,$ S.E.=0.094). In this model, the LRTs testing these two causal paths separately were both statistically significant: distal smoking → alcohol (NCP=5.77, df=1, p=0.016), and proximal alcohol → smoking (NCP=4.39, df=1, p=0.036). Parameter estimates from the best-fitting, restricted IV-CLPM model are shown in Table 4. For completeness, the parameter estimates from the full IV-CLPM model are shown in Appendix Table A2.

**Table 4.** Parameter estimates in the best-fitting IV-CLPM model applied to smoking status and alcohol use.

Parameter	Description	Estimate	*SE*	Wald’s *T*	*p*-Value
Regression paths					
bx1	*PGS of Smoking (Smk) → Smk at T1*	0.146	0.016	8.901	0.0000
bx2	*PGS of Smk → Smk at T2*	0.014	0.013	1.085	0.2777
by1	*PGS of Alcohol (Alc) → Alc at T1*	0.169	0.021	8.054	0.0000
by2	*PGS of Alc → Alc at T2*	0.037	0.018	2.036	0.0418
bx2x1	*Smk at T1 → Smk at T2*	0.808	0.022	36.461	0.0000
by2x1	***Smk at T1 → Alc at T2 (Distal)***	**0.065**	**0.027**	**2.404**	**0.0162**
bx1y1	***Alc at T1 → Smk at T1 (Proximal)***	**0.196**	**0.094**	**2.095**	**0.0362**
by2y1	*Alc at T1 → Alc at T2*	0.662	0.015	44.032	0.0000
(Co)variances					
Vpx	*Variance of the PGS of Smk*	1.000	0.020	49.472	0.0000
Cixiy	*Covariance of the PGSs*	0.196	0.015	13.441	0.0000
Vpy	*Variance of the PGS of Alc*	1.000	0.020	49.472	0.0000
Vx1	*Residual variance of Smk at T1*	0.726	0.040	18.248	0.0000
Cx1y1	*Covariance of residuals of Smk and Alc at T1*	−0.119	0.158	−0.751	0.4526
Vy1	*Residual variance of Alc at T1*	1.679	0.040	41.973	0.0000
Vx2	*Residual variance of Smk at T2*	0.179	0.010	18.803	0.0000
Cx2y2	*Covariance of residuals of Smk and Alc at T2*	0.047	0.013	3.611	0.0003
Vy2	*Residual variance of Alc at T2*	0.819	0.025	33.486	0.0000
Covariates					
b_smk_age	*Age → Smk*	0.007	0.001	8.026	0.0000
b_alc_age	*Age → Alc*	0.009	0.001	8.014	0.0000
b_smk_female	*Female sex → Smk*	−0.088	0.030	−2.974	0.0029
b_alc_female	*Female sex → Alc*	−0.812	0.039	−21.041	0.0000
Intercepts					
b0_resSmkPRS	*Intercept (mean) of the PGS of Smk*	0.000	0.014	0.000	1.0000
b0_resAlcPRS	*Intercept (mean) of the PGS of Alc*	0.000	0.014	0.000	1.0000
b0_smk1	*Intercept of Smk at T1*	−0.347	0.049	−7.007	0.0000
b0_alc1	*Intercept of Alc at T1*	3.818	0.062	61.984	0.0000
b0_smk2	*Intercept of Smk at T2*	−0.442	0.052	−8.466	0.0000
b0_alc2	*Intercept of Alc at T1*	3.580	0.065	54.752	0.0000

*Note.* Rows shown in bold indicate estimates of the causal paths between smoking status (latent liability scale of an ordinal variable with levels: never–former–current smoking) and alcohol use (drinks per week).

## Discussion

In this report, we confirmed the prior observation that the lagged Granger effects in the CLPM decay with increasing time intervals between study waves. We proposed the novel IV-CLPM approach to investigate causal effects when the CLPM’s lagged effects become undetectable at extended intervals. In the traditional CLPM, it is only possible to detect lagged effects within a narrow range of time intervals, and this window is primarily dependent on the autocorrelations of the two variables. On the other hand, in the IV-CLPM, the lagged Granger effects (i.e., the distal effects) are complemented by the IVR-based estimation of cumulative causal influences that have accrued over time (i.e., the proximal effects). Given the IVR-estimated proximal effects, the IV-CLPM allows us to infer causality even as the Granger-causal influences decay. Substantively, while the proximal effects help infer *whether* a predictor has a causal impact on the outcome, the distal effects would allow us to predict whether the effects of an intervention (on the predictor variable) would remain appreciable after a given time interval, with critical policy and practice implications. We also presented an empirical application, in which we examined bidirectional causal influences between cigarette smoking status and alcohol use (drinks per week) assessed three years apart. In this application, while the CLPM failed to reject the null hypothesis for a *distal* effect of alcohol use on smoking status, the IV-CLPM suggested that a *proximal* effect of alcohol on smoking fits the data better than the distal one.

### Causal inference at varying time intervals

The situation in which the proximal effect is significant while the distal effect is not (as was the case in our empirical analyses) sheds light on the relative importance of past and concurrent assessments of the causal variable. The significant proximal effect of alcohol use on smoking in our example is consistent with increased smoking urge and smoking behavior reported following alcohol consumption (Epstein et al., 2007; Mckee & Weinberger, 2013). At the same time, the non-significant distal effect suggests that this effect of alcohol use on smoking status fades away over time. Given these results, it is important to emphasize the distinction between the causal effect estimated based on the observed data (i.e., the “causal inference”) and the true, unobserved causal phenomenon. As discussed above, although the proximal effect is estimated as a cross-sectional path in the IV-CLPM, it does not necessarily imply that there is no temporal ordering between alcohol consumption (the cause) and smoking status (the outcome) in the true causal process. Rather, these findings suggest that alcohol consumption has a more immediate effect on smoking behavior, which is better approximated by a cross-sectional path than a lagged path over a time interval of three years. At smaller time intervals, this causal effect may indeed be best estimated as a lagged effect, as previously reported in a study using ecological momentary assessments (Piasecki et al., 2011).

Another possible example of this scenario is the study by Ma et al. (2019) of the relationship between anxiety and blood cortisol levels during early adolescence. Here, the authors found that social anxiety symptoms predicted blood cortisol levels assessed three years later, while physiological anxiety symptoms did not, even though the latter were concurrently correlated with cortisol levels. Adding IVs for physiological anxiety [e.g., genetic variants associated with anxiety (Levey et al., 2020)] to this model and estimating the proximal and distal effects in the IV-CLPM could help assess whether *recent* physiological anxiety symptoms have a causal influence on blood cortisol, even if the effect dissipates over three years.

Even when the time interval between study waves is too long for there to be a detectable distal effect, the IV-CLPM can be used to gain insights into how causal processes change over time. For example, if the underlying causal process is stationary, as the distal effect approaches zero at an extended time interval, the proximal effects at the two waves become approximately equal (Figure 4(B)). However, if the two proximal effects have different magnitudes (even as the distal effect is zero), that would indicate that the underlying causal process is not stationary. Applied in developmental cohort studies (i.e., where individuals from a specific age group are recruited and followed up longitudinally), the IV-CLPM could thus help assess whether the predictor’s effect (or the magnitude thereof) depends on the development stage at the two waves. As such, the IV-CLPM offers an attractive approach for identifying developmentally sensitive causal processes in studies, such as the *Adolescent Brain Cognitive Development (ABCD) Study^®^* (Garavan et al., 2018), the *National Longitudinal Study of Adolescent Health* (Add Health; Harris, 2013), and the *Twins Early Development Study* (TEDS; Rimfeld et al., 2019). These studies have also collected and genotyped DNA samples from the study participants, opening opportunities for using appropriate genetic variants as IVs for causal modeling.

In the IV-CLPM, the joint test of distal and wave-2 proximal effects, coupled with its comparison to the LRTs of these paths separately, provides a novel tool to examine whether the time interval between study waves is appropriate for testing Granger causality between two variables. Thus, for assessing causation between the variables of interest, evidence from the IV-CLPM can guide the selection of the most appropriate dataset (based on the time intervals in different available datasets). Here, it should be noted that the optimal time interval may differ for different pairs of variables, such that a time interval (in a given study with several variables) that is appropriate for estimating lagged Granger causality between variables *X* and *Y* may not be so for the causal effects between variables *X* and *Z*.

The unidirectional version of the IV-CLPM uses one IV (for the predictor variable *X*) to estimate the proximal and distal effects of *X* on *Y* (Appendix Figure A3). As the unidirectional model does not offer any power advantage over the full (bidirectional) IV-CLPM, the latter provides the more appropriate point of departure for model-fitting. If there is no evidence of feedback causal effects in the full model, the goodness-of-fit indices of the full model and the more parsimonious unidirectional model can be compared. The unidirectional IV-CLPM can also be the starting model if the *a priori* theory posits a unidirectional causal process.

### Application to smoking status and alcohol use

Our goal with this application was to demonstrate the empirical feasibility of the proposed model, as well as the etiological insights that this model might provide compared to the standard CLPM. Additionally, we demonstrated the utility of using genetic variants as IVs. For a detailed discussion of genetic IVs (i.e., *Mendelian Randomization* analyses), we refer the reader to previous review papers by Davey Smith and Ebrahim (2003), Lawlor et al. (2008), and Richmond and Davey Smith (2022), to name a few.

In our analyses, the CLPM indicated a statistically significant distal effect of smoking status on alcohol use (drinks per week), but the evidence for a reverse effect of alcohol use on smoking status was inconclusive. On the other hand, the IV-CLPM suggested a more complex bidirectional relationship involving a significant proximal effect of alcohol consumption on smoking status, in addition to the distal effect of smoking status on alcohol use found in the CLPM. Epidemiological studies have shown the concomitant use of alcohol and tobacco (Falk et al., 2006). Concurrent alcohol use is also reported to be associated with the transition from never smoking to (non-daily) current smoking (Campbell et al., 2012). Furthermore, high alcohol consumption is associated with a higher risk of smoking relapse (transition from former to current smoking), while low alcohol consumption is associated with a higher success of smoking cessation (transition from current to former smoking) (Weinberger et al., 2013). Our findings indicate that this relationship might be attributed, to some extent, to a causal effect of the quantity of alcohol consumption on smoking behavior. Experimental studies point to various potential mechanisms through which alcohol consumption may increase smoking behaviors, including increased craving to smoke and decreased ability to resist smoking (Mckee & Weinberger, 2013).

On the other hand, the distal effect of smoking status on alcohol use is consistent with the results of prior Mendelian Randomization analyses (Reed et al., 2022), as well as a U.S.-based longitudinal community study which also had a time interval of three years (Harrison & Mckee, 2011). Nicotine (from cigarettes) has been shown to reinforce alcohol reward (Mckee & Weinberger, 2013). Nicotine exposure may also counteract some of the negative neurocognitive effects of alcohol (e.g., subjective intoxication, cognitive impairment, and gait disturbance), increasing alcohol tolerance (Hurley et al., 2012). Both mechanisms could lead to increased alcohol consumption over time.

### Limitations

The three causal effects in the IV-CLPM provide unique information about the causal process that unfolded over different time windows. However, the power to test the three null hypotheses separately is relatively low, underscoring the need for large sample sizes. Although low power is a limitation of IVR generally, this problem inevitably worsens with increasing model complexity. In our empirical example, we observed larger standard errors of the causal estimates in the full IV-CLPM than in the restricted, best-fitting model (with the number of causal estimates reduced from three to one in either direction of causation; Appendix Table A2 *vs.* Table 4). Thus, even if the full model has limited power to estimate the three causal estimates precisely, it provides a useful point of departure when both proximal and distal effects are plausible (as was the case in our applied example of smoking and alcohol use).

As with the traditional CLPM, the IV-CLPM cannot distinguish the within-individual causal process from stable, between-individual heterogeneity (or the unmeasured time-invariant covariates). Doing so requires at least three repeated assessments and the introduction of a random intercept for each trait in the model (RI-CLPM; Hamaker et al., 2015). Second, like the CLPM, the IV-CLPM is a discrete-time model. In this model, it is assumed that all study participants have approximately uniform intervals between the two measurements, which may not always be the case. Alternative continuous-time approaches, such as stochastic differential equations (e.g., see Driver & Voelkle, 2018; Oud & Jansen, 2000), can overcome the need for equal intervals through direct modeling of the measured time intervals. Because of these limitations of the CLPM, future studies are planned to develop a random-intercept IV-CLPM model for panel data with three or more waves and to explore the integration of IVs into continuous-time longitudinal models. We discuss some of these potential extensions in the section on Future Research below.

Consistent with the IVR model (Bollen, 2012), careful selection of appropriate IVs is vital for robust causal inference in the IV-CLPM. As is true for all statistical models, the inference of IVR estimates rests on the validity of the model assumptions. Specifically, it is assumed that the IV influences the “outcome” variable only indirectly, exclusively through the path mediated by the causal variable (“exclusion restriction”). In other words, for estimating the effect of *X* on *Y* using the instrumental variable *IVx*, it is assumed that the residual variance of *Y* is uncorrelated with *IVx*. If this assumption is violated, the IVR-estimated proximal effects will be biased, and the bias increases with a larger covariance between *IVx* and the residual of *Y* (Maydeu-Olivares et al., 2019). In the proposed IV-CLPM, the two IVs are allowed to covary freely, which, in turn, allows the IV of one trait to covary with the other trait, independent of a direct causal effect between the two traits. In this case, *IVx* is a valid IV for estimating the (cross-sectional) proximal effects of X on Y in the IV-CLPM if the residual variance of Y ($E_{Y1}$ and $E_{Y2}$ in Figure 1(C)) is uncorrelated with *IVx* (i.e., over and above the sources of covariance between *IVx* and Y already included in the model).

The use of polygenic scores (PGSs) as IVs in our empirical application accommodates, at least to some extent, potential violations of this assumption, as the correlation between the two PGSs (due to shared SNPs) allows for the IV (*IVx*) to covary with the “outcome” trait (*Y*), independent of the causal effects between the two traits. However, to infer the proximal effect of alcohol use on smoking status in this application, we need to assume that there is no (unmodeled) covariance between the PGS of alcohol use and the residual variance of smoking status (which is not empirically verifiable). Moreover, it is assumed that the IV is not associated with any confounding variables affecting the predictor and the outcome (“exchangeability”). In our empirical application, the random segregation of genetic variants underpins this assumption. That said, as it is not possible to test all the assumptions of the selected IV in a particular empirical application (and thereby be certain that it fully satisfies these assumptions), consistency of the causal estimate in sensitivity analyses can help to add confidence to the causal inference. Therefore, to strengthen the evidence for the causal effects reported in our empirical application, it is advisable to perform additional sensitivity analyses of the validity of the IVs and other model assumptions.

For an empirical illustration of the proposed model, we operationalized smoking behaviors as an ordinal variable of smoking status (current *vs.* former *vs.* never smoking). However, smoking is a complex, multi-faceted construct with different etiological factors underlying smoking initiation, maintenance, heaviness, cessation, and relapse (Audrain-McGovern et al., 2015; Mahajan et al., 2021; West, 2017). Therefore, further research is needed for a comprehensive examination of the causal relationship between alcohol consumption and different aspects of smoking status (e.g., initiation, maintenance, and cessation) and heaviness (e.g., cigarettes per day).

### Future research and potential extensions of IV-CLPM

#### Models with more than two waves

In this report, we used the simplest case of CLPM for introducing the IV-CLPM approach, i.e., a model with two variables and two waves of data. This approach could be extended to the CLPM with three or more waves. However, for data with more than two waves, it is worth exploring if and how IVs could be integrated with alternative models that may address some of the other limitations of the CLPM (which, in turn, also apply to the IV-CLPM), as discussed in the previous section.

Adding IVs to a *random-intercept CLPM* (RI-CLPM; Hamaker et al., 2015) would allow differentiation of the causal process from stable between-individual differences, but would require data with at least three repeated assessments. In the RI-CLPM, the cross-lagged (causal) and autoregressive paths are modeled at the level of occasion-specific residuals (i.e., occasion-specific deviations from the individual-level mean). Conceptually, the random intercept controls for *unmeasured*, stable individual-level variance in that construct, while the IV controls for the *measured* individual-level variance. Likewise, the covariance of the two random intercepts reflects the covariance attributable to the *unmeasured* time-invariant confounders, analogous to the *measured* (time-invariant) genetic confounding captured by the covariance of the two PGSs in our empirical example.

Further, if four or more repeated assessments are available, an alternative random-intercept approach could be to incorporate IVs into the *dynamic panel model* (DPM; Allison et al., 2017). Although the initially proposed DPM included the causal paths in one direction only (say, *X → Y*), its recent iterations allow for bidirectional (i.e., cross-lagged) causal effects (Andersen, 2022; Murayama & Gfrörer, 2022). The DPM approach is argued to be less restrictive than the RI-CLPM and a more appropriate random-intercept model when the causal process in not stationary (Andersen, 2022).

Both the RI-CLPM and the DPM can help reduce the bias in the lagged causal effects, relative to the standard CLPM (and, by extension, the IV-CLPM presented here). However, a downside of using these alternative approaches as the base model for incorporating IVs into panel data would be that the power for estimating the causal effects (the primary parameters of interest) decreases with increasing model complexity and the number of estimated parameters from CLPM to RI-CLPM to DPM (Murayama & Gfrörer, 2022). The utility and feasibility of adding IVs to these alternative models is an important subject for future research.

#### Models with more than two variables

The IV-CLPM can also be extended to models with more than two variables, building on prior extensions of the CLPM. If multiple traits are hypothesized to have potential reciprocal causal influences on each other, such complex multivariate systems can be represented by the “mutualism” model, with pairwise bidirectional causal effects between variables (Borsboom et al., 2021; van der Maas et al., 2006). For repeated-measures data, this model has previously been extended as a CLPM with more than two variables, called the “dynamic mutualism” model, estimating pairwise cross-lagged effects between variables (Mcelroy et al., 2018). The dynamic mutualism model could also be seen as a less-restricted, bidirectional version of the unidirectional *auto-regressive mediation model* proposed by Maxwell et al. (2011). The latter involves three variables *X*, *Y*, and *M*, where *M* acts as a partial or complete mediator of the effects of *X* on *Y*. That is, *X* is allowed to have a lagged effect on *M*, which, in turn, may have a lagged effect on *Y*. In a model with partial mediation, *X* may also have a direct lagged effect on *Y*.

As in the standard CLPM, the lagged effects in these models will also depend on the time interval between assessments. Importantly, the time interval appropriate for estimating the lagged effects may differ across different pairs of variables in the multivariate system. Future research should examine the impact of time intervals on the causal inference in such multivariate models, as well as the pros and cons of adding IVs to these models to estimate proximal and distal effects. However, similar to the models with more than two waves, it will be important to examine the impact of increasing model complexity on the power to estimate the causal effects in these models.

#### Comparison with continuous-time models

Here, we have presented the impact of time intervals on causal inference in the CLPM and the utility of adding IVs within a discrete-time modeling framework (which also encompasses the RI-CLPM and the DPM). On the other hand, continuous-time models, such as those using stochastic differential equations (SDE), offer a more generalizable approach to estimate lagged causal effects in panel data (Voelkle et al., 2012). These models estimate the cross-lagged (and auto-regressive) effects per unit of time in the true continuous causal process (i.e., the derivative of these lagged effects with respect to time). As such, the estimated causal effects are not specific to the observed time intervals, thus overcoming the dependence of the lagged causal effects on the time intervals between study waves. The appropriate interval between discrete-time observations (i.e., the *sampling rate*) required for capturing the underlying continuous-time process is defined by the *Nyquist-Shannon sampling theorem* (Luke, 1999). If the *Nyquist-Shannon* criterion is not met in a study, the time interval may not be appropriate for fitting a continuous-time model. For an in-depth exposition of this theorem in the context of continuous-time modeling in behavioral sciences, we refer the reader to Voelkle and Oud (2013).

Future research should examine the utility of integrating IVs with continuous-time models in the SEM framework, especially when the *Nyquist-Shannon* criterion is not met in a study. This approach can offer additional advantages over discrete-time models, including accommodating non-uniform time intervals across study participants (Oud & Jansen, 2000), as mentioned above in the Limitations section. If three or more repeated assessments are available in a study, continuous-time models can also allow controlling for individual-level heterogeneity, akin to the discrete-time RI-CLPM and DPM approaches (Voelkle et al., 2012).

## Conclusion

The IV-CLPM can help address the ambiguity of non-significant causal estimates in the traditional CLPM. By estimating both Granger/lagged causal effects and the IVR-based proximal causal effects, the IV-CLPM can help detect causation even when the Granger-causal influences in the CLPM have decayed due to a long time interval. Thereby, the IV-CLPM can overcome the dependence of the traditional CLPM’s causal inference on the time interval between measurement occasions. Furthermore, the model provides a novel approach to examining whether the time interval in a study is appropriate for studying Granger-causal processes between a pair of variables. Finally, the proposed model also provides a flexible point of departure for exploring the integration of IVs with other longitudinal models, including models with random intercepts. To motivate empirical applications of the IV-CLPM, we have also illustrated the utility and limitations of using polygenic scores as IVs in large-scale panel studies with genetic data.

## Figures and Tables

**Figure A1.**
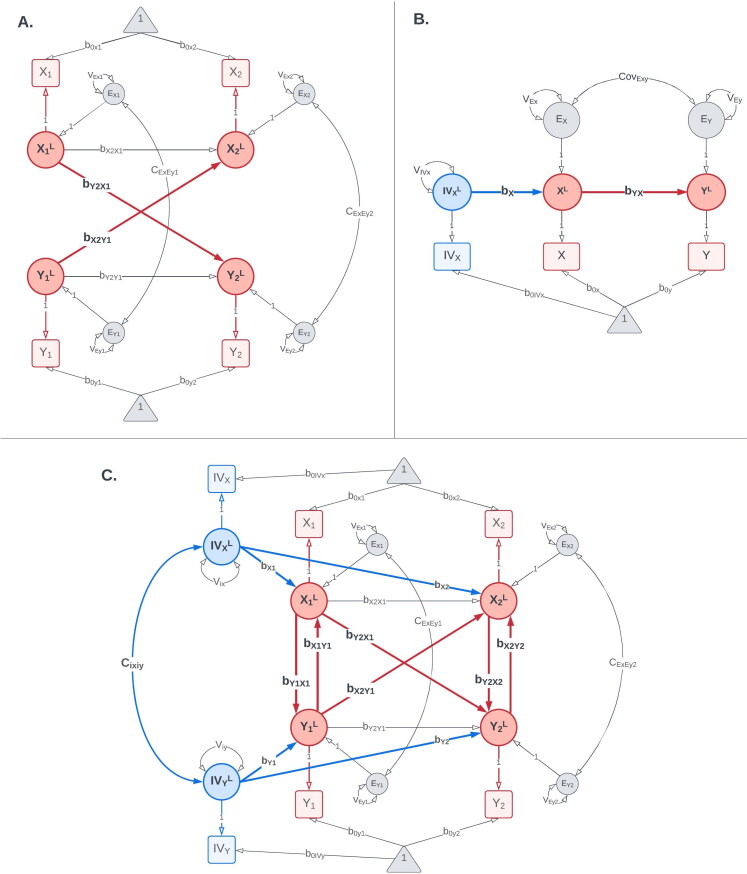
(A) CLPM (with means): The cross-lagged panel model (CLPM) is used to estimate bidirectional lagged effects between *X* and *Y* ($b_{Y2X1}$ and $b_{X2Y1}$). This model was used as the reference model for integrating instrumental variables. (B) IV Regression (with means): The instrumental variables regression (IVR) model fitted in a Structural Equation Modeling framework. The model uses the instrumental variable for *X*, *IVx*, to estimate the causal effect of *X* on *Y* ($b_{YX}$). (C) IV-CLPM (with means): The proposed IV-CLPM model combines the CLPM with bidirectional IVR applied cross-sectionally at each wave. In addition to the lagged (i.e., “distal”) effects $b_{Y2X1}$ and $b_{X2Y1},$ the model utilizes IVR to estimate cross-sectional (i.e., “proximal”) effects at each wave: $b_{Y1X1}$ and $b_{X1Y1}$ at wave 1, and $b_{Y2X2}$ and $b_{X2Y2}$ at wave 2. In all three path diagrams, squares/rectangles represent the observed variables, and circles represent latent variables. Triangles represent constants used to model the variables’ mean levels.

**Figure A2.**
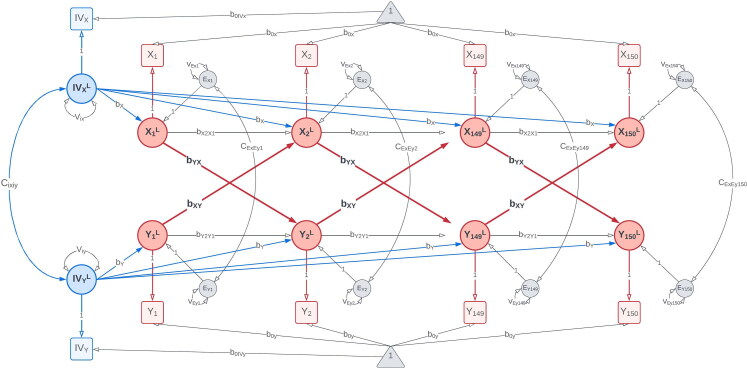
Data-generating model (with means): The data-generating model with bidirectional first-order causal effects between *X* and *Y* ($b_{YX}$ and $b_{XY}$) simulated over 150 time points. The instrumental variable for *X*, *IVx*, has an unchanging direct effect on *X* ($b_{X}$) at every time point. Likewise, the instrumental variable for *Y*, *IVy*, directly affects *Y* ($b_{Y}$) at all time points. Squares/rectangles represent the observed variables, and circles represent latent variables (i.e., the variances in this model). Triangles represent constants used to model the variables’ mean levels.

**Figure A6.**
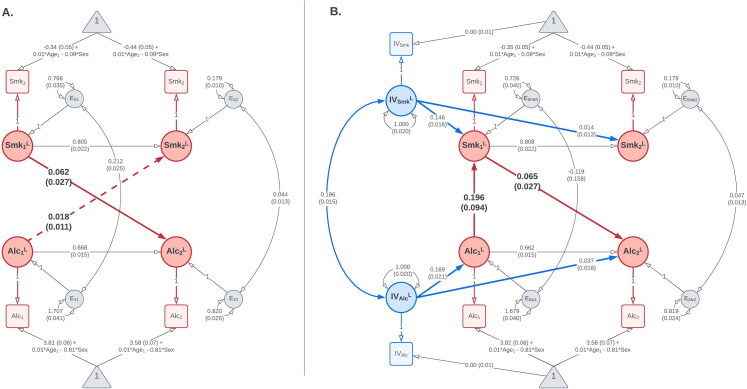
Results of (A) the CLPM (with means and covariates) and (B) the best-fitting IV-CLPM (with means and covariates) examining bidirectional causal effects between smoking status (Smk) and alcoholic drinks per week (Alc), assessed three years apart. The paths have been labeled with the point estimate and its standard error (in parentheses). The dashed path in the CLPM indicates a non-significant causal estimate. The CLPM suggests a likely unidirectional causal process, with a significant effect of smoking on alcohol use, but not *vice versa*. On the contrary, the IV-CLPM suggests a more complex bidirectional causation, with a significant proximal effect of alcohol use on smoking, which, in turn, has a reciprocal distal effect on alcohol use. In both path diagrams, squares/rectangles represent the observed variables, and circles represent latent variables. Triangles represent constants used to model the traits’ mean levels.

## Data Availability

Data from the Netherlands Twin Register (NTR) may be accessed for research purposes by submitting a data sharing request (evaluated by a data access committee) and signing a data sharing agreement. Further information about working with the NTR data is available at https://ntr-data-request.psy.vu.nl/.

## Article information

**Conflict of interest disclosure:** No potential conflict of interest was reported by the author(s).

**Ethical Principles:** The authors affirm having followed professional ethical guidelines in preparing this work. These guidelines include obtaining informed consent from human participants, maintaining ethical treatment and respect for the rights of human or animal participants, and ensuring the privacy of participants and their data, such as ensuring that individual participants cannot be identified in reported results or from publicly available original or archival data.

**Funding:** This work has been funded by the U.S. National Institutes of Health (NIH) grants R01DA049867 (MCN, CVD, HHMM, MS) and 5T32MH-020030 (LFSC). MCN was also supported by funds from the Rachel Brown Banks’ Chair of Psychiatry at Virginia Commonwealth University. DIB was supported by the Royal Netherlands Academy of Arts and Sciences (KNAW) Academy Professor Award (PAH/6635). ANTR Survey 8 was funded by ZonMW (Addiction) Project: 31160008 (PI: DIB). (ZonMW is a partnership between ZorgOnderzoek Nederland (Care Research Netherlands, Dutch abbreviation: ZON) and the Medical Sciences (Dutch abbreviation: MW) domain of the Dutch Research Council (NWO).) ANTR Survey 10 was funded by ERC (European Research Council) Starting Grant 284167 (PI: JMV).

**Role of the Funders/Sponsors:** None of the funders or sponsors of this research had any role in the design and conduct of the study; collection, management, analysis, and interpretation of data; preparation, review, or approval of the manuscript; or decision to submit the manuscript for publication.

**Acknowledgments:** We are grateful to the twins and their families participating in the Netherlands Twin Register (NTR). We also thank the anonymous reviewers for their insightful feedback on the previous versions of this manuscript.

## Code availability statement

Code for reproducing the simulations presented in this paper is available on GitHub here https://github.com/singh-madhur/IV-CLPM_manuscript.

## Appendix A

**Table A1.** Pearson’s correlations between the model variables in one of the simulated datasets, showing some of the time points used for model-fitting.

	IVx	IVy	X100	X101	X125	X150	Y100	Y101	Y125	Y150
IVx	1									
IVy	0.2500	1								
X100	**0.2834**	**0.2227**	1							
X101	**0.2834**	**0.2227**	0.8490	1						
X125	**0.2834**	**0.2227**	0.1581	0.1640	1					
X150	**0.2834**	**0.2227**	0.1087	0.1092	0.1581	1				
Y100	**0.2227**	**0.2834**	**0.6877**	0.6904	0.1532	0.1038	1			
Y101	**0.2227**	**0.2834**	0.6904	**0.6877**	0.1591	0.1042	0.8490	1		
Y125	**0.2227**	**0.2834**	0.1532	0.1591	**0.6877**	0.1532	0.1581	0.1640	1	
Y150	**0.2227**	**0.2834**	0.1038	0.1042	0.1532	**0.6877**	0.1087	0.1092	0.1581	1

*Note*. The simulated time-series was stationary over the time points used for model-fitting (i.e., from T=100 to T=150), as indicated by the correlations and between the instrumental variable (*IVx*) and *X_i_* (r=0.2834), *IVx* and *Y_i_* (r=0.2227), and the cross-sectional correlations between *X_i_* and *Y_i_* (r=0.6877). The shown correlations are based on a time-series with the direct effect on *IVx* on *X*, $b_{X}=0.08;$ the direct effect on *IVy* on *Y*, $b_{Y}=0.08;$ the first-order autoregressive coefficient (AR1) for *X*, $b_{X2X1}=0.7;$ the AR1 for *Y*, $b_{Y2Y1}=0.7;$ the first-order causal effect of *X* on *Y*, $b_{YX}=0.2;$ the first-order causal effect of *Y* on *X*, $b_{XY}=0.2;$ the cross-sectional correlation between the residuals of *X* and *Y*, $r_{\mathrm{exy}}=0.2;$ and the correlation of *IVx* and *IVy*, $r_{IV}=0.25.$

**Table A2.** Parameter estimates in the full IV-CLPM model applied to smoking status and alcohol use.

Parameter	Description	Estimate	*SE*	Wald’s *T*	*p*-Value
Regression paths					
bx1	*PGS of Smoking (Smk) → Smk at T1*	0.144	0.017	8.335	0.0000
bx2	*PGS of Smk → Smk at T2*	0.014	0.013	1.084	0.2783
by1	*PGS of Alcohol (Alc) → Alc at T1*	0.160	0.023	7.077	0.0000
by2	*PGS of Alc → Alc at T2*	0.036	0.019	1.910	0.0561
by1x1	***Smk at T1 → Alc at T1 (Proximal)***	**0.158**	**0.143**	**1.108**	**0.2680**
bx2x1	*Smk at T1 → Smk at T2*	0.809	0.032	25.202	0.0000
by2x1	***Smk at T1 → Alc at T2 (Distal)***	**0.303**	**1.071**	**0.283**	**0.7774**
bx1y1	***Alc at T1 → Smk at T1 (Proximal)***	**0.200**	**0.100**	**1.996**	**0.0459**
bx2y1	***Alc at T1 → Smk at T2 (Distal)***	**0.083**	**0.233**	**0.358**	**0.7208**
by2y1	*Alc at T1 → Alc at T2*	0.669	0.027	24.449	0.0000
by2x2	***Smk at T2 → Alc at T2 (Proximal)***	**−0.298**	**1.326**	**−0.225**	**0.8223**
bx2y2	***Alc at T2 → Smk at T2 (Proximal)***	**−0.100**	**0.349**	**−0.285**	**0.7757**
(Co)variances					
Vpx	*Variance of the PGS of Smk*	1.000	0.020	49.472	0.0000
Cixiy	*Covariance of the PGSs*	0.196	0.015	13.441	0.0000
Vpy	*Variance of the PGS of Alc*	1.000	0.020	49.472	0.0000
Vx1	*Residual variance of Smk at T1*	0.729	0.043	17.146	0.0000
Cx1y1	*Covariance of residuals of Smk and Alc at T1*	−0.244	0.198	−1.234	0.2174
Vy1	*Residual variance of Alc at T1*	1.633	0.046	35.566	0.0000
Vx2	*Residual variance of Smk at T2*	0.196	0.089	2.208	0.0272
Cx2y2	*Covariance of residuals of Smk and Alc at T2*	0.181	0.365	0.495	0.6205
Vy2	*Residual variance of Alc at T2*	0.861	0.260	3.312	0.0009
Covariates					
b_smk_age	*Age → Smk*	0.007	0.001	8.046	0.0000
b_alc_age	*Age → Alc*	0.009	0.001	8.018	0.0000
b_smk_female	*Female sex → Smk*	−0.088	0.030	−2.978	0.0029
b_alc_female	*Female sex → Alc*	−0.812	0.039	−21.034	0.0000
Intercept					
b0_resSmkPRS	*Intercept (mean) of the PGS of Smk*	0.000	0.014	0.000	1.0000
b0_resAlcPRS	*Intercept (mean) of the PGS of Alc*	0.000	0.014	0.000	1.0000
b0_smk1	*Intercept of Smk at T1*	−0.346	0.049	−7.007	0.0000
b0_alc1	*Intercept of Alc at T1*	3.819	0.062	61.955	0.0000
b0_smk2	*Intercept of Smk at T2*	−0.444	0.052	−8.511	0.0000
b0_alc2	*Intercept of Alc at T1*	3.579	0.065	54.707	0.0000

*Note.* Rows shown in bold indicate estimates of the causal paths between smoking status (latent liability scale of an ordinal variable with levels: never–former–current smoking) and alcohol use (drinks per week).
